# Psychological Quarterly Retrospect

**Published:** 1856-04-01

**Authors:** 


### *Psychological Quarterly Retrospect.*

[By the Editor.]

The preceding quarter has been replete with important psychological *data.* We have endeavoured to seize upon some of the more important salient points, with a view of permanently placing them upon record in this department of the journal, as illustrations of the social, moral, and religious progress of the age. The subject of crime, as a type of epidemic disorder, is fully considered in another part of the *Psychological Journal.* To this elaborate article we earnestly direct the attention of our readers. The subject is one of deep and philosophic interest.

During the preceding quarter the much-vexed question of capital punishment has again been brought under public notice and discussion, by Mr. Bright, in an able and spirited speech, recently delivered by that honourable gentleman to his Manchester constituents. He states the grievance of those who oppose the punishment of death with his usual acuteness, sagacity, and vigour of illustration. We must, however, confess that we are not made a convert to his opinions. What other punishment could be substituted in lieu of the gallows for the crime of murder at all calculated to protect society? We have uniformly expressed strong opinions against all undue severity of punishment, and have never failed to protest against the existing inequality between certain criminal offences and the punishment generally awarded for their commission; but for the crime of murder, what punishment short of death upon the scaffold would meet the exigencies of the case? But let Mr. Bright speak for himself.

"If I meet with a man who argues in favour of hanging, and listen to his conversation, I find that he uses exactly the same arguments that were used thirty years ago in favour of hanging for all those offences that Mr. Dymond has described. I remember reading a speech of Sir Fowell Buxton's in the House of Commons, in which he said that if a young man murdered his grandfather, or if he cut down a young tree, then the law imposed just the same penalty for each offence. Some years ago, more than 200 offences were punishable with death. A bloody and a horrible system of law grew up chiefly since the Revolution of 1688; and during the time the present family have been on the throne of this country—and we have been under the government of an aristocracy assumed to be very enlightened, and a Government much more free than we had before that Revolution—more than 200 offences, such as the stealing of a fowl, or of 5s. worth out of a shop, were punishable with death. Only conceive how perverted men's minds must have become by this common custom of hanging to tolerate such things. I recollect hearing of the case of a woman whose husband had been kidnapped by the pressgang, and she stole something from a shop in Ludgate-hill. Well, for the sake of example, and to preserve the property of Ludgate-hill shopkeepers, she was hanged, and nobody at that time was shocked at the matter. I have read the memoirs of Elizabeth Fry—an excellent person, whom I consider it a happiness that I have been acquainted with—in which she describes that for the issuing of a bad half-crown, or a bad pound-note, persons were in Newgate in great numbers; and out of twenty or thirty convicted, the custom was to select a certain number—three or four, or eight or ten—and doom them to the gallows. This was common—at that time almost every week, in London—and the people passed by Newgate every week, and saw human beings strung up, swinging in the breeze, and separated after the execution; and the great statesmen of that generation never asked scarcely whether this horrible custom might not be mitigated or abolished. The world, however, was not all in that darkness. There was one country in the world where the law was not so brutal,—a great country on the other side of the Atlantic, springing up from colonies founded by ourselves. In the year 1675, William Penn founded the state of Pennsylvania—that is, in the reign of Charles II. Consider how long ago that is. Now we are accustomed to fancy that we have made prodigious strides in science and everything else since that time; but when William Penn drew up the laws of Pennsylvania, assisted by the great and good men who were his helpers, they did not carry with them the 200 capital offences we had here, but struck them all out, with the exception of one, that being the one we are now chiefly discussing—namely, capital punishment for murder. They retained that punishment for only aggravated cases of murder; and Penn is understood to have retained it only in deference to the wishes of others, and not from any belief in its necessity or propriety. The State of Pennsylvania is now a great commonwealth, and during the whole of the time that has elapsed since its foundation, no person has been put to death in it for any crime save aggravated murder, and for even aggravated murder in extremely few cases. But your great statesmen that you read of in your histories—the men that shone in the House of Lords and the House of Commons—what were they doing then? Why, extending this law for scores of offences; and since William Penn's time, many thousands have been hanged in this country for offences that never were capitally punished in Pennsylvania, and for which the punishment of death has since been abolished without the slightest injury to any one. All these persons, if the law were as rational as it was in Pennsylvania, would not have been executed, and I am satisfied that it would have been of enormous advantage to the country if it had been so, for it is impossible to bring a human being into a street, in front of several hundred persons, and hang him, with all the indignity that you would treat a dog with, without doing damage to the moral sense of every man and woman who shall gaze on that horrid spectacle. While such was the state of things in Pennsylvania—I take that merely as an example, for there are other countries I might mention—men were hanged in this country needlessly; and during all that period you had great authorities for what you did. I recollect dining in Dublin some few years ago, and sitting at table next to a judge on the Irish bench. I was discussing this question with him, and in the course of our conversation he said to me, 'Never take the opinions of judges on a question of that nature. They are accustomed to the thing. They are brought up to look upon it from a particular and peculiar point of view. If judges only had been consulted, you would probably still be hanging for horse stealing and sheep stealing, as you were a few years ago.' I am happy to say there are, and have been, of late years, not a few judges, and some of the highest in character and reputation, who have been quite willing that the punishment of death should be abolished; and we may expect that the experience they receive, if they are men of ordinary enlightenment, must bring them more and more to that conclusion. But governments do not like to part with any power, and they do not like to part with any power over the lives of their subjects. For offences against the Sovereign or the State, look at the horrible punishment the law enacts. Hanging, cutting up, tearing out the heart, drawing, quartering, every description of cruelty, was, until within a very short time since, the law; and a portion of these horrors is still the law with regard to offences that go under the name of high treason. These persons don't examine this question. Politicians are quite willing to do nothing except what they are driven to, and to make their game of politics in things as they are, if the people do not force a change."

Mr. Bright much questions whether the punishment of death has at all diminished the crime of murder.

"One argument, and a most potent one, in convincing me that hanging was a blunder, is this; and I ask your attention to it for one moment. What we all want is, to make human life as secure as possible, and there are two modes by which it is proposed to effect it. One is by punishment by the law—the law hopes to deter and frighten men. The other is by inculcating everywhere a deep reverence for human life. Does anybody in England believe that human life is not more secure in England now than it was 100 or 200 years ago, when there was far more hanging than there is now? The reason why we feel more secure is because we know there is a less reckless, savage, and brutal character among all classes now than there was then; that there is more politeness, gentleness, courtesy, benevolence, and kindness of every description now than at former periods. There is a greater belief, arising from religious knowledge, that human life is a thing of infinite estimation in the eyes of the Creator, and it is this greater reverence for it which is the great and, I hope, the growing security for human life. I will assume—but I doubt it—that the practice of hanging men has the effect of deterring somebody, in some place, at some time, in some degree, from the crime of murder. How many do we save by reason of hanging, and how many are saved by reason of an increased reverence for human life? Don't you think that the reverence for human life that exists more or less in everybody, is a thousand times more effective in preventing murder than the fear of the gallows? But if it should happen that the system of hanging, however it may deter to some small extent, does to a great extent diminish the reverence for human life—don't you think that what it does in deterring is far less than what it does in destroying, by diminishing the sanctity of human life? I am perfectly certain—I know from my own feelings and from all human history—that participation in horror of any kind familiarizes the mind to it. Don't we know that when the news of the battle of the Alma was received, people shuddered when they read the account of the losses of that short but bloody battle? Don't you know that now we can read of losses far greater without being affected by them? It is so with everything of the kind, and so with public executions. That man who murdered his master in Drury-lane some time ago was a case in point. The very day that man was apprehended, I happened to be at the police-office in Westminster, where I went with a gentleman, now a member of the House of Commons, to give notice of a person who had run away with 200*l*. or 300*l*. belonging to him. In the office there was a policeman, more intelligent than many of that class, who addressed me by name. I asked, 'How do you know me?' 'I know you very well,' said he; 'have you never seen me in the lobby of the House of Commons?' 'Yes, I recollect you now,' I said, adding, 'You seem to have had some very bad cases latterly; I see a lad has committed a murder.' He said, 'Ay, sir, as long as there's hanging there will be murders.' I thought that this would have been excellent for a judge, and I wanted to know how he got to that conclusion. 'They don't care about it,' he said. 'Why, what did Weeks do (that was the name of the man who committed the murder)? He went to see a man hanged at Newgate in the morning. He ran at half-past nine o'clock the same morning to see a woman hanged at Horsemonger-lane, and what he said was this, 'Why, it's nothing—it's but a kick, and it's over in a minute.'"

But, as the Editor of the *Examiner* asks, how can Mr. Bright's theory of capital punishment be brought in harmony with his practice? "How is it that he is found a frequent applicant at the Home Office for the commutation of the sentence of death? If the convict thinks so little of the scaffold, and so much of the secondary punishment, how can Mr. Bright be so cruel as to use his influence and powers of persuasion to get the man subjected to the severer and more dreaded penalty? Surely the Home Secretary has henceforth a reply out of his own mouth for him. 'Why, Friend Bright, after all, no one knows better than you, who have profited by the experience of letter D No. 1, that it's only a kick.'

"Yes,' our friend would rejoin, 'it is only a kick, but it is a last kick, and no one likes to do anything, no matter how trifling, for the last time.'"

The point in dispute in a great measure hangs upon this question—does the fear of death deter from the commission of capital offences? does it operate as an effective restraint upon the murderous propensities of others? That it does so to a certain degree there can be no doubt. Many a man is deterred from sacrificing human life, even under extreme provocation, and with certain stringent penalties and great severity of punishment staring him in the face, influenced solely by the love of life and the fear of suffering a painful and ignominious death upon the scaffold. There is a certain type of criminals, devoid of all moral sense, having neither the fear of God or man before their eyes, who, reckless of all consequences, commit the most atrocious crimes. No amount of ingenious torture, or certainty of punishment in perspective, would deter a particular class of criminals from the commission of capital offences. It is not, however, with small sections of society that legislation has to deal: the laws cannot be so framed as to mete out even-handed justice to exceptional cases. The criminal code must be general in its operation; and there can be no exemption from punishment, except in cases of clearly-established moral irresponsibility, associated with positive mental aberration. The Editor of the *Examiner* maintains that the chances in favour of the worst guilt, have increased, and are alarmingly increasing. He says:

"The technicalities of the law give the murderer his chances; the spurious sensibility of jurors and the crotchets of abolitionists give him his chances; and the influences which operate at the Home Office give him his chances. See how much he has to calculate upon. First, he may not be found out; secondly, if found out, he may not be convicted. Is there not a Wilkins at the bar, and may there not be a Quaker on the jury? Thirdly, if convicted, is there not a Home Office where a man with active friends may have his case brought under review with all the pleadings on the side of indulgence?—we use not the word mercy, for mercy regards the safety of the many, and will not expose them to danger to spare the guilty.

"The consequence of all this is, that criminal justice is affected with the very highest degree of uncertainty, and crime proportionately encouraged. A man who meditates murder now is not scared by the thought that the capital penalty follows the crime as its very shadow. *Il y a fagots et fagots.* There are murders and murders. There are discriminating penalties on the varieties. There is indulgence for murder without aggravating circumstances. Who knows till he has tried what will be considered aggravation, what not? It is all a lottery, and with few bad billets. The most select of all circles is now the halter. What a man must do to get hanged now-a-days is something prodigious. He has, as it were, to pass a competitive examination without parallel for difficulty. And for the deserving candidates their name is legion."

The suggestive and demoralizing effect of *public* executions on the criminal population is a point entirely apart from the question of the abolition of capital punishment. The mischief resulting from public executions is undoubtedly great. The fact that so many thousands rush with eagerness to see a fellow-creature strangled to death is in itself sad and melancholy evidence of the existence, in a considerable portion of our population, of a very questionable state of moral sense. We subjoin the following account of a public execution in California, and ask our readers whether such a revolting spectacle was not calculated to demoralize the mind, vitiate the feelings, and harden the hearts of those who witnessed it?

"The execution of two men, named Crane and Free, took place recently in California, which was attended by upwards of 5,000 persons. The *Placewell* gives the following account of the revolting affair:—'At one o'clock, p.m., accompanied by Sheriff Carson and his assistants, the prisoners came forth and took their seats quite gaily in a waggon. Crane was elegantly dressed in a full summer suit of white—pants trimmed with blue, a blue sash, with rosette or star upon his breast, and looked and acted the perfect Kentucky gentleman. Free was also well dressed; light coat, trimmed with red; a red sash; black pants, trimmed with red; an elegant low-crowned black rowdy hat, which he wore a little to one side, and appeared not unlike an unconcerned jolly sailor. Arriving at the foot of the gallows, both ascended with a firm step to the platform. Free quite gaily (both being entirely unconfined) skipped upon the drop and round the platform, smiling upon the arrangement. They were now permitted to address the assemblage. Crane, in a very gentlemanly manner, and apparently without the slightest hesitation, read in a loud, firm voice his address. He seemed to regret nothing, except that he had not been permitted to take his own life long before; that he was soon to be with his beloved bride, but who the world called his murdered victim, and continued to speak for nearly twenty minutes. Free said he had concluded not to make any remarks, as it would only excite the people present, and stepped back from the edge of the scaffold. Calls from the crowd to go on! let us have it! brought him forward. He then said, 'I'll give you Micky Free's scaffold song;' commenced, but after singing three lines, stopped, saying, 'Gentlemen, I can't go through with it; you must excuse me.' Crane came forward and commenced singing a hymn from manuscript, probably of his own composition, in which Free heartily joined. They were clothed in the habiliments of the grave, and stepped upon the drop without the slightest apparent reluctance. The ropes were adjusted about their necks, a prayer offered up to the God of heaven and of earth for mercy on their souls, and with the 'Amen' fell the drop and the two murderers. The last words of Free were: 'Now, boys, see that this is done right.' Crane's last words were: 'Susan receive me; I will soon be with you.' Crane was tried and found guilty of the murder of Miss Susan M. Newenham, near Ringgold, El Dorado county. He shot her with a Colt's revolver. Free had, according to his confession, and the evidence adduced on his trial, led a long career of crime.'"

Let us turn from these disgusting details to a published account of a triple execution in Indiana. We place the facts upon record in order that they may form material for future psychological reflection. We live in an age of advancement—moral, social, religious, and political progression; and important facts like those just referred to may constitute *data* for grave legislative consideration:—

"Three men, named Rice, Driskill, and Stocking, were recently hung at Lafayette, Indiana; the scene is thus described in a local journal:—At twelve o'clock they were asked if they were ready for dinner. Rice replied, 'Yes; I am hungry.' Driskill said that he wanted 'a good old dinner, as it was the last;' he didn't 'want to die hungry.' He remarked to Rice, 'We'll get supper somewhere else, Abe.' The dinners were brought in and despatched with great heartiness. After dinner each of them in turn washed and dressed himself for the final moment. They could not have made their toilet with more deliberation and coolness if they had been going to a frolic. Driskill, when washing, remarked through the window, near which he was standing, to some one outside, that he was 'getting a good ready.' In putting on his shirt, Rice discovered that there was a button missing. Driskill told him to sew one on; Rice replied that he hadn't time. Driskill nonchalantly rejoined, that there was 'an hour yet!' He complimented Rice with looking 'd—d starchy.' Rice, as he finished, observed, 'Well, gentlemen, I reckon there never was a willinger soul to die than I am.' Stocking said nothing, but conducted himself (as he did throughout) with dignity and firmness. He was dressed in a blue frock coat, black cloth pants, black satin vest, boots, and hat, presenting a very respectable appearance. When he was done and his arms had been pinioned, he sat down on a bench and picked up a newspaper, which he composedly read until the preparations of the others were completed. Rice was dressed in two pairs of drawers, the outward pair being woollen knit and fitting close, and shoes. He wore neither coat nor vest. Driskill wore nothing but shirt, pants, and socks. Before they were led out to the scaffold, they passed around the cells and bid adieu to the other prisoners. Stocking presented one of them with some shirts and a handkerchief and hat, and to another a Bible and Prayer-book. He vouchsafed to them all a word of advice, telling them to avoid bad company and liquor, and to treat their parents, if they had any, well; he had never done justice to his. At two o'clock they were brought upon the scaffold, which, as we have previously described, was erected at the north-east window of the court-house. Some thirty or forty persons were within the enclosure. Rice, on entering, addressed them with 'How do you do, gentlemen?' and on recognising a number of acquaintance, called them up and shook hands with them. They were then asked by the sheriff if they had anything to say. They then each delivered an address, protesting their innocence, but stating they died willingly. The sheriff proceeded to adjust the fatal ropes. Rice requested that a stool which had been placed for his accommodation on the scaffold might be removed, and on his request not being immediately complied with, he removed it himself. He then knelt down, inclined his head forward, remarking that he had 'seen men hung,' by which we understood that he regarded that as the proper position. Driskill on observing it, said, 'Abe, are you going to kneel?' Rice answered 'Yes.' He then turned to Stocking and said, 'Stock, which way is the easiest to die—kneel or stand? I want to die the easiest way.' Stocking replied that he should stand, unless there was danger of the rope breaking. The sheriff assured him there was no danger. He therefore stood up, but Driskill kneeled. The caps were then drawn over their faces, and at twenty-two minutes and a half after two o'clock the bolt was withdrawn, and the culprits launched into eternity. None of them gave evidence of protracted suffering.'"*

Can scenes like these elevate the moral character of a nation? Are they not calculated to call into active exercise the most depraved and ferocious of human passions and instincts? It is a great, a fatal mistake, to imagine that the dross, the refuse of the population of great cities, that are usually found congregated in the immediate precincts of the scaffold, are at all elevated in the social scale by the sickening sight of a fellow-creature dangling in the convulsive agonies of death at the end of a rope. Such exhibitions, even when conducted with the greatest consideration for public feeling, only tend to brutalize the mind and paralyze the better aspirations of the heart. We subjoin a passage from the "History of a Wasted Life," that speaks trumpet-tongued on the subject:—

"I had been many hours on Waterloo-bridge. And now I discovered that passengers across it, going towards the Strand end, became frequent. Singly and in groups passed hurriedly by me hundreds of poorly-dressed men, women, boys, and girls—all pushing forward to one point. One would have imagined from their jokes, as they hurried by, that something mightily amusing was in prospect. I was not long in doubt as to what it really was.

"'Wonder wot he's doing on't now!' said one of the youths.

"'Tom 'll die game, I know,' remarked one of the girls. 'He's a regular brick. Lord! how he used to go and see the hangings hisself!'

"'Come along,' remarked another of the party, 'or we sha'n't get nigh the drop;' and the whole of them closely wrapping their rags about them, joined the eager crowds who were still hurrying towards, as I now knew, the Old Bailey, in which stands Newgate prison.

"I soon learned from the passers-by that a young man was to be executed within eight hours from that time—Monday is always the hanging day in London—and hence this unusual travel over the bridge at midnight.

"It was fine overhead, and though by no means partial to such sights, I resolved to go to the Old Bailey. In the bustle and excitement, I thought the dark hours would pass less wearily away, and so I followed, and made one of the multitude, which now, in dense masses, thronged up Ludgate-hill. . . .

"Six weeks before the period to which I am now especially alluding, I was compelled, in the exercise of my newspaper duties, to attend at the Old Bailey in order to furnish particulars of the execution of a woman. The wretched creature about to suffer had been a servant to an old lady in Westminster, whom she had strangled in order to procure a five-pound note—which note, after the crime was committed, the murderess discovered to be a counterfeit one.

"In order to have a good place, I secured a seat in the window of a public-

* *Times*, Feb. 6, 1856.

house directly opposite the scaffold, for which I, or rather the proprietor of the journal, had to pay five shillings. At about an hour before the time of execution, then, I got with much difficulty through the crowd, and established myself in my position.

"I had not long been seated, when another individual entered the room. He, too, I saw, paid five shillings—a sum which I should not have thought, from his appearance, he could have at all afforded. He was a young man of apparently eighteen or nineteen years of age, and bore the appearance of a worker at the anvil. He had a low, sulky, hangdog sort of a look, his eyes were bloodshot, and a great heavy under jaw gave an animal expression to his countenance, and a short, thick, bull-neck, conferred on him quite the stamp of ferocity. A short black pipe was stuck in his mouth, and in his hand, as he entered, was a jug of 'dog's nose,' a compound of beer, gin, and nutmeg. This he set down on the sill of the open window, and then sprawling on a chair, he began whistling an air which was at that time remarkably popular among the ragamuffins and rascals of London.

"'Drink, mister,' said the blacksmith, pushing his jug towards me. The invitation, however, I respectfully declined, the consequence of which was, that I evidently sunk many degrees in the young fellow's estimation. He was glum and silent for some time, but at length he knocked the ashes from his pipe, and said:—

"'By ——! only think of the girl's being scragged for a bad flimsy. Curious, now, aint it?'

"I said something or other, I forget what, and then he became quite communicative about the hangman; it was evidently a favourite topic of his.

"'I knows Calcraft well enough; I have smoked many's the pipe with him. He's a shoemaker up in the New North-road, just out of City-road. Meet 'im in the street, and he's dressed so spicy you'd take 'im for a reglar swell.'

"'Indeed,' I remarked.

"'Yes,' said he, 'a hout and houter.'

"'You seem fond of this sort of thing,' I hinted.

"'I just am, and no mistake. Vy, I ha'n't missed a hanging this five year —what d'ye think o' that, old feller?'

"Being on the spot myself just then, I could not with a very good grace say much in depreciation of his taste, so I held my tongue."

"It was positively awful to see how he gloated over the spectacle. As the moments flew he actually became impatient, and cursed the time that it did not fly faster—it was rapid enough for the poor victim within. When St. Sepulchre's bell commenced tolling eight, he leaned from the window, his eyes glaring with excitement, and his frame actually quivering with joyful anticipation. A cheer actually burst from his lips when the gaol-door opened, and the criminal, pinioned and pale, tottered forth. As his practised eye marked her emotion, he ground his teeth and cursed her for her 'want of pluck.'

"No sooner had the drop fallen, than he hurried from the room. I remained to pen my notes, and when I went down stairs half an hour after, the execution hunter was sitting, surrounded by a select circle of admiring listeners, to whom he was relating his hanging experience in general, and his impressions respecting the affair of that morning in particular. He was very much intoxicated, and, glad to escape his recognition, I hurried away.

"And now, reader, let me take up the thread of this narrative just where I snapped it a few moments ago.

"When I reached the end of the Old Bailey, on Ludgate-hill, the crowd became so dense that it was with the utmost difficulty I could force my way through the somewhat narrow street toward the well-known 'debtors'-door' of Newgate. All along the road to this place, the taverns were opened, and doing a thriving business. The gin-palaces were thronged, and coarse jokes respecting the coming dreadful event were heard in all directions, for Hang-Monday was a holiday in the calendar of crime. It seemed that on this particular occasion, more than usual interest was felt in the criminal. He was an apprentice who had killed his master whilst intoxicated with drink and passion, and the crime had been perpetrated in the most daring manner, in the very heart of London. Thousands of apprentices swelled the mob, and most of them seemed to think that they were doing honour to Tom Wicks (for that was the victim's name) by attending his execution.

"Just as two o'clock sounded from the church steeples, a bustle was seen in front of the prison, and a huge wooden machine was drawn out from the great doors. Men then began to work by the light of torches, and yell after yell of savage satisfaction was heard as the different portions of the scaffold were put together. And (will it be believed?) when the upright and the cross-beam, constituting the gallows, were raised and fixed, one huzza burst from the crowd. Some women, with children in their arms, indeed, shuddered and turned pale; but the jeers of their brutal companions speedily banished all feeling, and they became as fierce and foul as others. Many of the persons present had come provided with bottles of spirits, and whenever a poor, shivering infant wailed or wept with cold, or screamed from the pain caused by pressure, it was dosed into quiet by gin. And fearful was it to see how those infants sucked in the poison—that poison which caused the gibbet to be erected, and which, in the fog and mist, loomed up before the eyes of men and women who, even then, were preparing for it new victims.

"Three—four—five o'clock; and the crowd became terrific. I stood close to the barriers, and at times the pressure of the enormous host behind me was fearful. At length, by a mighty wave, as it were, of that sea of humanity, I was dashed forward, taken off my feet, and borne I know not whither. Instinctively I clutched at a lamp-post as I was being carried past it, and holding firmly on, the current swept by, and left me safe in an elevated position.

"At length seven o'clock came, and with it the grey light of morning; and what a spectacle did that light reveal! Not a spot within sight but was covered with haggard, anxious, staring humanity; and all in that crowd had come to see a fellow-creature die in mortal agony. But none appeared awed; the very boys seemed to enjoy the scene.

"As the appointed hour—that of eight—drew nigh, still greater became the anxiety. At length the bell of St. Sepulchre's commenced tolling one—two—three—(cries of 'Hats off')—four—five—six—seven—eight.

"And before the last bell-note died away, the debtors' door opened, and the buzz of twenty thousand tongues died away into silence. Then came the doomed man forth, preceded by a clergyman, and followed by the executioner and other officials.

"From my raised position, I could, without any intervening obstacle, gaze on the criminal as he stood under the beam. Possessed with a horrible curiosity, I leaned forward as far as possible, and, just as the miserable wretch lifted his head to take a farewell glance at the world—his eyes met mine. I fancied he started—I knew he did—for we recognised each other.

"Another instant and the white cap was drawn over the pale, wild face; a dull "thud" was heard as the rope tightened, and the quivering form of the murderer dangled from the gallows.

"He was the very same young man who with me sat in the tavern opposite, but six weeks before, gazing on the gallows which had now terminated his existence. Strangely enough, too—from that very tavern he had rushed, whilst intoxicated, for the purpose of shooting his master in Drury Lane; and in that same tavern the officers of justice found him when they sought his capture a few hours afterwards."

We cannot altogether omit to refer to one singularly remarkable and important sign of the social progress of the age, which has developed itself during the preceding quarter. The times certainly cannot be considered "out of joint," when a noble, wealthy Marchioness, of high and exalted descent, and educated in an ultra-political school, feels it to be her duty to leave the walks of private and domestic life, and assume the position of a public lecturer. It is not difficult to conceive what would have been the sentiments of the late Marquis, if he could have revisited the "pale glimpses of the moon," and have listened to the able address of the Dowager Marchioness of Londonderry, replete as it was with good sense, wisdom, generous, kind and liberal sentiments. During the delivery of this able address, which was particularly directed to the homely sympathies of her hearers, the Marchioness was interrupted by frequent manifestations of loud applause. At the termination of the speech, the audience rose in a body, and gave their fair and noble orator three hearty British cheers. The assembly consisted of nearly four thousand men, employed in her ladyship's collieries, on Chilton-moor. The entertainment consisted of a very liberal and substantial feast. A finer body of men than those three thousand sturdy pitmen who turned out at the bidding of their noble mistress could not have been assembled in any agricultural district in the kingdom. They all looked stout, well-fed, and merry; had clean, shining faces; were well clothed, and in intelligence were certainly superior to many working men who are conventionally looked upon as their superiors. The Earl Vane occupied the chair, and after the usual preliminaries, solicited, on behalf of his mother, the attention of the meeting for a few moments. Her ladyship, at first, spoke in a voice tremulous with emotion, but, as she warmed with the subject, her confidence in herself gained strength, and she proceeded with emphasis and marked feeling to address the meeting as follows:—

"My friends,—I have invited you all to dinner to-day that we may become better acquainted—that you may hear from my own lips the assurance of the deep interest I feel in your welfare, and that I may have an opportunity of expressing the pride and satifaction I have in presiding over so large a body of intelligent and well-conducted people. I have asked all in my employ to participate in this feast, and I bid you a friendly welcome. But I address myself principally to the pitmen, many of whose fathers worked under my ancestors. It is pleasing to trace back this bond of union, which I fondly hope may extend to the next generation, and that their children may continue to serve under mine. I regret that since the management of these great concerns has devolved on me I have not had health or strength to visit you underground as I could have wished. Indeed, I have never been able to persuade Mr. Elliot to promise to take me down, and I am afraid I should not succeed in finding my way alone. But I hope nevertheless, I may be permitted to say without vanity or presumption, that no colliers are more carefully looked after. Your comforts, your homes, and your schools, have been anxiously watched over. The latter have long enjoyed a proud pre-eminence; and although I have refused to place them under Government inspection and supervision, I know that they are well managed, and it is your own fault if you take your children away too soon and thus deprive them of the benefit of the good education that is provided for them. You well know how necessary that is for success in after life. We need not travel beyond the precincts of this building for instances of persons who have been the architects of their own fortune. It is the pride and boast of your head viewer that he was reared and nursed a boy in these pits, and it must be encouraging to look around this great mining country and see the many instances of men who have won their way to wealth and fame by labour and perseverance. On the other hand, I am proud to say you have set an example to the whole trade. You were the first to return to the old-fashioned system of 'binding,' and you have worked on steadily when the men in adjoining collieries were on the strike. You have seen them turned out of their homes—their furniture lying on the roads, and they and their belongings seeking shelter, while you have been comfortable by your own hearths in your peaceful homes, enjoying the reward of honest industry. Long may this happy state continue, and may you ever feel how much your interests are entwined with those of your employer. It is a subject of great thankfulness that these collieries have been for some time spared and exempt from any serious accident. Casualties will sometimes occur, notwithstanding all the precaution and vigilance of overmen and your viewers. And here let me endeavour to impress strongly upon your minds how much depends on your own prudence and care. I would I could find words or had eloquence to make this warning emphatic, for, I regret to say, the reports I receive of recklessness fill me with pain and alarm, and I know that Mr. Elliot has lately had occasion to visit most severely some fearful instances of negligence with the safety-lamp, that might have caused the most terrific results. You see that, although I have not been down, I am well informed of what passes below. You are all aware of the circumstances I allude to, and that those careless people have been punished, some by law, some by dismissal. Let me implore, I beseech you, that you will be careful and watchful, and remember, each of you, that not only your own lives, but those of hundreds, hang upon a thread—the gauze of your lamps—the shutting or the opening of a door. And while I ask for God's blessing on your undertaking, fail not to do all in your humble power to deserve it. I advise you to frequent and support your reading rooms, your mechanics' institutions, your temperance societies, and to avoid the public-house—to be orderly, industrious, and religious. I speak not of men's creeds,—they rest between man and his Maker. Nor do I presume to dictate on this momentous subject beyond expressing my anxious hope that you will hallow the Sabbath, and each attend your own place of worship. That duty paid, you will better enjoy the rest and recreation the Sunday afternoon should bring for yourselves and your families. (Cheers.) As a friend of religious liberty, I have not refused sites for chapels of different persuasions, where the numbers have justified my doing so, and I hope, in a few months, the church at New Seaham will be finished and available for the mining population residing there. I wish you were nearer to Wynward, which was lately visited by 600 members of the Stockton Mechanics' Institute, who applied to me for permission to see the house. They expressed themselves highly gratified, and were most orderly, quiet, respectable, and well-conducted. And now, my friends, I will not detain you longer than to tell you how much pleasure it has given me to see you all here, and to express my hope that you will remember and heed my words. I feel deeply the responsibility of my position, and I have thought it right to advise and counsel you to the best of my humble power; and I hope that while I am permitted to preside over you, we may each, individually and collectively, continue to endeavour to do our duty in that state of life to which it has pleased God to call us."

It is not our intention to consider, in this part of our journal, the many important points involved in the disputed question relative to the strictly religious observance of the Sunday. We may on a future occasion discuss this matter in detail. We now only allude to the subject with a view of quoting an extract from a beautiful Latin poem, published by his grace the Archbishop of Canterbury, when a scholar of Eton, in 1798, upon the words, *Parvus Deorum cultor et Etonensis.* This poem has recently been published in a fasciculus of "Musæ Etonenses," by the learned Provost of King's College, Cambridge. The poem is a bucolic, in which two parties, Mopsus and Amyntas, respectively advocate the duty and ridicule the superstition of religious observances on the Sunday. The passage referred to deserves to be permanently placed upon record:—

"MOPSUS.

"Non equidem invides: audivi quod in urbe profanis,
Tunc quoque, cum meliora audire et discere fas est,
Luditur ac bibitur: choreis epulisque vacantes
Vel proceres, aiunt (at vix ego credulus) ipsos
Septima lux frustra vocat ad delubra! Quis olim
Proteget heu miseros, si sint ea vera, Britannos?
Ast egomet grates refero, si videt agellus;
Sui, auctore Deo, cadit adversissimus annus,
Ut modo, cum thalami consortem Baucida febris
Abripuit, geminosque mihi contagia tauros,
Non queror impatiens, spero at meliora precorque.
Ast alii potius taurivi, vel altera conjux
Ante diem pereat, quam flectere genera negantem
Ludentemve notent redeuntia Sabbata Mopsum!
Nam, si forte greges, hac luce aut jugera curo,
Nescio quid sub corde dolet, nec tristius angor,
Cum ruit infestus mediis in messibus imber."*

At the recent Reading Assizes, a boy of twelve was indicted for murdering a boy four years old. The boys had been seen together, cutting furze, and the body of the younger one was found in a pit. On being questioned, the elder boy, who had gone home and behaved as though nothing whatever unusual had occurred, very coolly denied having seen the other, or having had anything to do with the matter: spoke of him as "a poor little fellow whom he knew very well, and was very sorry for;" adding, that he must be a very hard-hearted man who could so knock about the head of such a child. On finding that there was evidence to prove some of his statements false, he changed his tactics, and said that while he was cutting the furze the bill-hook came off the handle and struck the child, and that he became so frightened at what he had done that he killed him. This version Baron Bramwell and the jury appear to have kindly accepted, and the boy was acquitted! The Judge did indeed go so far as to give

* *The Churchman.*

him an admonition, telling him that whenever any accident happened to us it was always best to go and confess it, and not seek to cover it by doing worse things, &c. &c. Not a single witness to character was called, and a local paper intimates that such witnesses would not have promoted an acquittal. Why the boy should not have been indicted also for manslaughter, on his own version of the case, we cannot comprehend, for we suspect that his acquittal was mainly owing to the reluctance to condemn so young a boy to death. On the acquittal being known in the locality of the murder, a crowd of persons assembled at the railway station to meet the young murderer—for such we have no doubt that he is—and it was with difficulty that he could get home. The crowd then gathered round the cottage, broke the door and windows, and would have proceeded to further violence had not the father come out and promised that he should be removed out of the parish by daybreak. This satisfied them, and the promise was fulfilled by the boy proceeding to a neighbouring town or village; but we believe that he was ejected from that place also, and is about to be "sent to sea." Was not this boy known to his neighbours as one quite capable of such a crime? If not, would they have acted thus? The only mystery in the matter is the motive for such a crime, and of this no explanation is given.

There have been many strange rumours afloat, both in Paris and London, relative to the mental condition of the late Emperor of Russia. The Paris correspondent of the *Manchester Guardian* relates the particulars of a ghostly "warning" that the late Czar is said to have had in 1854. The statement is a curious and interesting one; and, without vouching for its authenticity, we lay it before our readers:—

"Towards the last days of January, 1854, (my informant being then at the French embassy in St. Petersburg,) a most extraordinary story was whispered about in the *salons* of the Russian capital, and was to the effect that the Czar Nicholas, alone in his study, saw all at once before him a monk of gigantic stature, of whom instantly the Emperor asked, 'what he wanted.' The monk said he wanted to talk with the Czar upon the war he was about to rush into. The Czar said that was not his business; whereto the monk answered, 'I have come to warn you. Remember this: if you are really undertaking this war for pure motives, and for the glory of the orthodox Church, you will succeed; but, if not—if you are obeying the dictates of your ambition, you will fail; and not only will your armies be vanquished, *but you yourself will come to an untimely end before the close of the war!*' The Czar, exasperated, rushed at the monk, and called the guards from the waiting-room. The guards came in, and found the Czar clutching at thin air, and apparently in a great rage, and constantly calling out to them—'The monk! the monk! who among you has seen the monk? Stop him! prevent him from escaping.' Here was the history as it circulated stealthily in the *salons* of St. Petersburg, in the last days of January, 1854."

The melancholy suicide of Mr. John Sadleir, M.P. for Sligo, has cast a dark cloud over the metropolis, and has afforded material for much newspaper comment and animadversion. It seems that the deceased was at his club up till half-past ten on Saturday night, at which hour he returned home to his residence, 11, Gloucester-square. At the club his friends observed nothing strange in his manner, and when he arrived home he seemed in his usual state of mind. He then ordered coffee; and as he required nothing further, the servants, as was usual with them at that hour, retired to rest, leaving their master up drinking his coffee. Whether he went out immediately after partaking of the coffee, or remained in until early the following morning, and then strolled up to Hampstead, no one can say; but it is clearly certain that he did not go to bed that night, as his bed was found undisturbed the following (Sunday) morning, and when the servants got up they found their master absent. It seems that he was in the habit frequently of staying several days at Hampstead, his friends stating that he invariably resided at Jack Straw's Castle Tavern.

When the deceased was found he was lying, as if asleep, on a small mound, which he had evidently picked out for the occasion, and a silver cup, together with a large-sized bottle, marked with several labels, "Poison," by his side. Life had been extinct some time, although the body was then warm. Inspector Green had the body removed to the workhouse, and, upon examining the deceased's pockets, he found a small slip of paper, on which was written, in a clear bold hand, "John Sadleir, 11, Gloucester-square, Hyde Park." There was also some money in gold and notes in the deceased's pockets, as also a case containing two razors, and several lumps of loaf-sugar, which the deceased, no doubt, contemplated taking the poison in. Inspector Green, through the slip of paper alluded to, was at once enabled to send to town and have the deceased identified, the writing on the paper being recognised as his own; so that the ill-fated gentleman must have prepared it with the express intention of its being the means of leading to his identification. The silver cup was also immediately recognised as the deceased's own property, through its bearing his crest, a "lion rampant." The dispenser's name and address was on the bottle, "John Maitland, 10, Chesterfield-terrace, Hyde Park-square;" and Inspector Green has been enabled to ascertain that the deceased sent to Mr. Maitland a written communication by his maid-servant, who brought the bottle containing the poison back to her master on the Saturday evening. The servant believes that a sovereign was paid for the poison, so that there is little doubt but that the bottle was full when supplied by Mr. Maitland. The deceased was a bachelor.

The extent of frauds and forgeries committed by the late Mr. John

Sadleir has not been ascertained; but there is every reason to believe that it will not be much under 1,000,000*l*.

It has already been ascertained that Mr. Sadleir had forged no fewer than 50,000 Royal Swedish Railway Company's shares and obligations for 5*l*. each, on which large sums of money were raised. This forgery alone is to the nominal sum of 250,000*l*. It is not yet known what amount he was able to raise on the shares and obligations.

In addition to these forgeries, there are forgeries of several deeds and mortgages of estates in Ireland. As regards these, however, the extent is not yet known.

The boldest and most daring forgeries of all are those which relate to deeds for the purchase of property in the Encumbered Estates' Court of Ireland. These forged deeds purport to bear the signatures of the commissioners, the registrar, the chief clerk, the solicitors in the various causes, and the commissioners' seal. The extent to which this class of forgeries has been committed may be inferred from the fact, that the stamp duty alone on the deeds amounted to several hundreds of pounds.

A considerable number of forgeries on private individuals have been discovered, and there is every reason to believe that others yet remain to be ascertained. Those which have been detected are supposed to amount to nearly 100,000*l*.

In addition to the classes of forgeries already enumerated, Mr. Sadleir has been guilty of the assignment of deeds, held in trust by him, to an enormous amount.

The drafts of all the forged deeds and mortgages are in Mr. Sadleir's own handwriting. The forgeries of signatures are, in every case, remarkably successful. Those of the Encumbered Estates' Commissioners are said to be so perfect that the most experienced eye could not detect the forged deeds from the genuine.

The act of self-destruction was, it is believed, precipitated by the circumstance of one of the holders of deeds relative to the purchase of one of the encumbered estates, an attorney in this country, who had advanced 10,000*l*. to Mr. Sadleir, having just gone over to Ireland with his solicitor for the purpose of getting the deed registered. Mr. Sadleir endeavoured to frustrate the intention of the party, but failed. Hence, it is presumed, the commission of suicide at the particular time it took place—longer concealment of his crimes being seen to be impossible.

On Saturday afternoon, at five o'clock, Mr. Sadleir met a large speculator in the City, Mr. * * * *, it is said, as he was in the act of starting for home. He shook his City friend cordially by the hand, and said to him cheerfully on parting, "Good bye; I am going to make a long journey, and we may not meet for some time."

It has been forcibly observed by a contemporary, that the "last scene of Mr. Sadleir's life was in keeping with his whole career. As he stood alone in the darkness of the midnight hour upon that solitary heath, at the portals of eternity, and saw the world for which he had sacrificed so much shrivel up like a parched scroll, the agony of the moment must have been too bitter to imagine. At the same time, it may truly be said of John Sadleir, that nothing in his life became him so much as leaving it. He perished in his prime, a criminal of the first magnitude,

"And left a name at which the world grows pale,
To point a moral and adorn a tale."

At the coroner's inquest the jury unanimously recorded a verdict of *felo-de-se.* Mr. Wakley's address on the occasion was marked by his usual good sense, shrewdness, ability, and eloquence. The question involved is one of such deep interest to the psychologist that we should ill discharge our duty if we omitted to place a full report of Mr. Wakley's masterly charge to the jury before our readers. After recapitulating the salient facts of the case, and referring to the mischief resulting from the common practice of returning verdicts of insanity, as "a matter of course," in every case of suicide, Mr. Wakley observes:

"The practice has this pernicious effect,—that jurymen, getting into the habit of returning such verdicts in cases where there are no crimes apparently charged, subsequently have to go to the Old Bailey and try cases of murder, and recollecting on what slight grounds they have returned verdicts of insanity before, every now and then, by this practice, the worst of criminals altogether escape the hands of justice, and the real madman gets executed. Nothing, therefore, can be more unfortunate than such investigations as to the state of mind of the person who commits the act of self-destruction. Mr. Manning has alluded to an issue directed by the Court of Chancery. I am not at all sorry that he has done so. Because, what happens? There are twenty-four intelligent jurymen, eight or ten medical practitioners, six or eight remarkably clever counsel, and sometimes half a dozen solicitors. They sit from day to day, conduct their investigation with the utmost assiduity, inquire into the state of mind of the party who is before them. By the ingenuity of counsel, the brain of the party is, as it were, rasped for the purpose of ascertaining its quality. Questions of the most searching character are put to the individual who is supposed to be insane. His manner is observed. The catenation of his ideas or the breaches in the links of the chain of connexion of his ideas are looked into. But in such cases the jury have the opportunity of seeing and hearing all that transpires in relation to the individual. They can exercise their own sense by observing his conduct, and the concretions of his mind. They also hear the elaborate opinions of medical men. And what frequently is the result? Why, at the end of the inquiry, four or five medical men believe the party to be sane, and four or five believe him to be insane. The counsel entertain the opinions which they did when the inquiry commenced, and there is only a very small majority of the jury on one side of the question.

If that be the condition of a court inquiring into the state of mind of a person in their presence, what is our condition when an individual has entirely passed from us, and when we can only form an opinion from such fragments of evidence as he has left behind him? Mr. Manning has alluded to the consequences of your verdict. Now, I am bound to tell you, that you must not take the consequences of your verdict into consideration at all. If you do, you may be altogether misled, and not guided by the evidence. The inquiry and the verdict are legitimate, and are the limits of your duty, and you must not consider the consequences in coming to your decision. In going from the dead back to the living individual, we must begin with the last link in the chain of life, as far as it is before us in evidence. It appears that the last person who saw Mr. Sadleir alive was the butler, who says he left him at eleven o'clock at night, and that at a quarter to one in the morning he found three letters on the hall slab, two of them addressed to Mr. Keating and one to Mr. Norris. He then ascertained that Mr. Sadleir had taken his greatcoat and hat and left the house. After that time we are not aware that Mr. Sadleir was seen alive by any person that knew him. The butler says he had lived with Mr. Sadleir eighteen months—that he was a man of sober and frugal habits, very temperate indeed in his mode of living—that he only took a glass or two of wine at dinner, and that no wine was put upon the table afterwards. The truth is, that every person who knew Mr. Sadleir, was well aware that he was an exceedingly temperate man. I have sat in the same room with him myself, and it was scarcely possible to find a man of more moderate and rational habits in ordinary society. The butler states that during that eighteen months he had not known Mr. Sadleir attempt to commit suicide, or threaten to commit suicide, and that he had observed nothing peculiar in his manner during the whole period he lived with him; that he had observed no change in him within the last month, fortnight, or week. He stated that a few minutes before seven o'clock on the Saturday evening, Mr. Sadleir requested him to go to Mr. Maitland's for the essential oil of almonds, and that at nine o'clock Mr. Sadleir rang the bell and asked if that had come—and that he placed it by the side of Mr. Sadleir without Mr. Sadleir making any remark. It seems that during the evening the butler had noticed that Mr. Sadleir was more hurried in his manner than usual, and that having got into a cab on one occasion he ran back into the house again, and that on another occasion when a cab had crossed the square he came back and ran up stairs, but that usually he was a cold and self-possessed man. At a quarter to eleven Mr. Norris left Mr. Sadleir, having been with him about half an hour. Mr. Norris stated that he observed a redness about Mr. Sadleir's eyes as if he had been weeping, and that when he arrived Mr. Sadleir was walking about the room, which he said was a very unusual occurrence. Whilst Mr. Norris was with Mr. Sadleir a telegraph message was brought in from the Reform Club, to the following effect:—'Telegraph message from James Sadleir, Merrion-square, to John Sadleir, Reform Club, Pall-mall, London.—Dublin Station.—"All right at all the branches; only a few small things refused here. If from £20,000 to £30,000 over here on Monday morning, all is safe."' Probably at that time Mr. Sadleir knew the impossibility of remitting so large a sum of money. But it appears that the poison had been sent for three hours before the arrival of that message. It also appears from the evidence of Mr. Norris that he had been with Mr. Sadleir on the afternoon of that day at Mr. Gurney's office, and Mr. Norris stated that he was so impressed with the effects likely to be produced on the mind and feelings of Mr. Sadleir by the reverses he had met with, that he should not be surprised if he shot himself. That was the remark made by Mr. Norris in the course of the afternoon, and it showed that something had occurred to impress Mr. Norris's mind with the conviction that there was the possibility in that case of suicide. Now, after Mr. Norris left Mr.

Sadleir's house, at a quarter to eleven, the letters addressed to Mr. Keating and Mr. Norris appear to have been written. These letters therefore contain the latest evidence we have as to the condition of the unfortunate gentleman's mind. Mr. Manning says he would have you attach little importance to the declarations in these letters, but they are presented to you in the character of dying letters. Mr. Manning says, and says truly, that Mr. Sadleir might have been in a state of great depression of mind in consequence of the losses he had met with, and the disasters which had occurred. That might bring you to the consideration of the question, 'What is insanity?' It is not depression of spirits. Insanity is not remorse—insanity is not agony of mind. I am sorry to say there is not a single sentence on record which gives an accurate definition of what is insanity in the eye of the law, and I fear such a definition will never be given. It is a subject which has engaged the attention of some of the ablest thinkers who have ever lived, psychologists of the highest reputation; but the whole question is environed with difficulties in consequence of the chameleon character of the varieties of insanity, and the causes of insanity. The one are as numerous as the other. If insanity could be defined by the words grief, or sorrow, or remorse, or even despair, greatly as insanity exists now, it would exist then in a tenfold, a thousandfold greater degree. But what you have to look to with reference to the evidence is this. Do you believe that at the time Mr. Sadleir committed the act of self-destruction he was a responsible agent—in other words, that he was in such a condition of mind as made him morally and legally responsible for his actions? You have to ask yourselves this question, and you have to refer to the evidence for the answer you must give in recording your verdict. It is very much to be regretted that the subject is one of so much difficulty, especially as these inquiries have to be instituted. But I have always seen through my experience that jurors are disposed to lean to the side of mercy and humanity. It should, however, be borne in mind that if there be mercy and humanity with reference to the individual, the same thing refers more largely to society; and it is on this point that I think the practice of returning verdicts of insanity, and temporary insanity, where the evidence is slight, is operating most disastrously in this country in our criminal courts of justice, and I trust the day is not far distant when such a change will be made in the law as will entirely put an end to that practice. Now, you have before you in evidence the fact that Mr. Sadleir had sustained great losses, and in his own communications he states that he has committed great crimes."

Mr. Wakley then laid the following correspondence before the jury as important evidence of Mr. Sadleir's state of mind just prior to the commission of suicide:

"'Saturday night.

"'I cannot live; I have ruined too many. I cannot live and see their agony. I have committed abominable crimes, unknown to any human being; they will now appear to light, bringing my family and others to distress—causing to all shame and grief that they should have ever known me. I blame no one, but attribute all to my own infamous villany. . . . . And hundreds of others are ruined by my villany. I could go through any torture as a punishment for my crimes. No torture could be too much for such crimes, but I cannot live to see the tortures I inflict upon others.—J. Sadleir.——Telegraph to —— and otherwise when you read.'

"In that note all the sentences are accurately placed, and it does not betray the least confusion of ideas. Then comes the first note addressed to Mr. Keating:—

" 'Dear Robert,—James sent me his title-deeds of the Coolmanack and Kilkonnell estates. I have not used these deeds in any way. I gave J. Gurney a letter from James, entrusted to me by him, which J. Gurney had sent to him. This letter cannot be acted on by J. Gurney without my brother's express authority.

" 'R. Keating, Esq., M.P.' " 'John Sadleir.

" 'February 16, 1856.

" 'T. Uzielli has a bank bill for 2000*l*., on which nothing is due. It should be at once cancelled. If on Monday the bank is to be saved, 8200*l*. must be paid to the East Kent Railway for two orders—6200*l*. and 2000*l*. 2500*l*. must be paid into Glyn's, to meet an order at sight issued to-day at Carrick. Gurney knows the orders falling due on Tuesday are all advanced, save the one for 6200*l*. in my favour.

" 'This must be taken up on Monday next, being advised. I cannot live. " 'J. S.'

"When he concludes this note on all these matters of business, he says, 'I cannot live.—J. S.' His mind is not then in such a state of disturbance even as to create a confusion of ideas; but matters of business are accurately stated and represented to the gentleman whom he addressed. The next letter is also written to Mr. Keating. The hour is not stated, but it is one of the letters left on the hall slab:—

" '1, Gloucester-terrace, Feb. 16, 1856.

" 'Dear Robert,—To what infamy have I come, step by step, heaping crime upon crime! and now I find myself the author of numberless crimes of a diabolical character, and the cause of ruin, and misery, and disgrace to thousands—ay, of tens of thousands!

" 'Oh, how I feel for those on whom all this ruin must fall! I could bear all punishments, but I could never bear to witness the sufferings of those on whom I have brought such ruin. It must be better that I should not live.

" 'No one has been privy to my crimes. They sprang from my own cursed brain alone. I have swindled and deceived without the knowledge of any one. Stevens and Norris are both innocent, and have no knowledge of the fabrication of deeds and forgeries by me, and by which I have sought to go on in the honeyed hope of retrieving.

" 'It was a sad day for all when I came to London.

" 'I can give but little aid to unravel assets and transactions.

" 'There are serious questions as to my interest in the Grand Junction and other undertakings.

" 'Much will be lost to my creditors if these cases are not fairly treated.

" 'The Grand Junction, the East Kent, and the Swiss line, the Rome line, and the Coal Company, are all liable to be entirely lost now, so far as my assets are concerned.

" 'I authorize you to take possession of all my letters, papers, property, &c., in this house, and at Wilkinson's, and at 18, Cannon-street.

" 'Return my brother his letters to me, and all other papers. The prayers of one so wicked could not avail, or I would seek to pray for those I leave after me, who will have to suffer agony, and all owing to my criminal acts.

" 'Oh, that I had never quitted Ireland! Oh, that I had resisted the first attempts to launch me into speculations!

" 'If I had had less talents of a worthless kind, and more firmness, I might have remained as I once was, honest and truthful, and I would have lived to see my dear father and mother in their old age. I weep, and weep now, but what can that avail? 'J. Sadleir.'

"Then comes the letter addressed to Mrs. James Sadleir:—

"'James is not to blame. I alone have caused all this dreadful ruin.

"'James was to me too fond a brother, but he is not to blame for being deceived and led astray by my diabolical acts.

"'Be to him at this moment all the support you can.

"'Oh, what would I not suffer with gladness to save those whom I have ruined!

"'My end will prove, at least, that I was not callous to their agony.'

"Now, in these letters, written at almost the last moment before Mr. Sadleir died, there is nothing inconsistent with the perfect retention of the reasoning and reflecting powers, and also the maintenance of a correct memory. At the same time, it is impossible not to know and feel that from the very manner in which the letters are expressed, he must have been suffering the most intense agony. The very act which he committed must of itself prove the mental suffering which drove him to such a measure of desperation. But still, again arises the question, was he in that state of mind which made him irresponsible —was he aware of the character of the act he was about to commit—was he driven to it by an uncontrollable impulse that entirely weighed down his reasoning and reflecting powers—or did he know perfectly well what he was doing, and had the power of controlling his actions, and of controlling them to such a degree as to make him responsible? These are the questions which you will have to answer by your verdict. In the examination of the bodies of persons who die from suicide, we generally wish the most careful research to be made into the state of the brain. That examination appears to have been made in this case by Mr. Nicholl, but it appears that no disease of the brain was found to exist, nor was any disease found in the membranes of the brain. There was a slight effusion at the base of the skull, but nothing to which any great consequence can be attached. There was no inflammatory action going on in the brain, although the membranes were rather more congested than usual. Mr. Nicholl found no sufficient disease to lead him to believe there was any malady affecting that organ. Sometimes in the heads of persons committing suicide, extensive disease is found, sufficient to account for insanity. Although disease was not found, disease to a slight extent may have existed, and it may have escaped observation in consequence of the peculiar texture of the brain itself. You will weigh everything which has been adduced in the shape of evidence—even the allusion to the opium. There can be no objection to your taking that into account. Mr. Nicholl believes that he found particles of opium in the stomach. But it does not appear that Mr. Sadleir had purchased opium, or that he was an opium eater, or had been affected by the use of that drug, and there was no indication of it in his manner on the Saturday night. But you will take all the evidence which has been adduced into your consideration. If you believe he was of unsound mind and was irresponsible for his actions—that he was so far driven by an impulse to commit the act, and committed no moral offence in so doing, you will say he was of unsound mind at the time he took the poison. But if, on the other hand, you believe he was of perfect memory and understanding at that time, and that he knew perfectly well what he was doing, and that he could have controlled his act if he had thought proper to do so, you can come to no other conclusion than that he committed murder, and murdered himself whilst in a sane state of mind. If you believe the contrary, you will say he was of an unsound state of mind. If you have any doubt on the subject, I would call on you to give his memory the benefit of that doubt; but if you have no doubt, it is impossible you can come to any other conclusion than that it was an act of self-murder.

"The jury then retired, and after an absence of about twenty minutes, returned into court, when the Foreman said: We are of opinion that the deceased died by his own hand when he was in a perfectly sane state of mind.

"The Coroner: Are you unanimous?

"The Foreman: We are unanimous.

"The Coroner: My own opinion is, after the most mature and deliberate consideration, that you could have come to no other conclusion.

"The inquisition was then signed in the usual manner, and the proceedings terminated."

It is much to be regretted that a verdict of *felo-de-se* should, in these enlightened times, be considered necessary in order to satisfy the demands of justice. In the case of Mr. Sadleir, we agree with Mr. Wakley, that there was no positive and conclusive evidence before the court to establish the insanity of the deceased. The jury might, however, have escaped from the difficulty, and have protected the bereaved family of this unhappy gentleman from further obloquy and disgrace, by adopting a common practice, of returning, what is termed, an *open verdict*. Verdicts of *felo-de-se* can lead to no good, satisfactory, or practical result.

We are but now re-echoing opinions expressed by ourselves in print sixteen years ago, at which period we thus record our sentiments in respect to verdicts of *felo-de-se*:*

"The act of suicide ought not to be considered as a crime in the legal definition of the term. It is not an offence that can be deemed cognizable by the civil magistrate. It is to be considered a sinful and vicious action. To punish suicide as a crime is to commit a solecism in legislation. The unfortunate individual by the very act of suicide places himself beyond the vengeance of the law; he has anticipated its operation; he has rendered himself amenable to the highest tribunal, viz., that of his Creator; no penal enactments, however stringent, can affect him. What is the operation of the law under these circumstances? A verdict of *felo-de-se* is returned, and the innocent relations of the suicide are disgraced and branded with infamy, and that too on evidence of an *exparte* nature. It is unjust, inhuman, unnatural, and unchristian, that the law should punish the innocent family of the man who, in a moment of frenzy, terminates his own miserable existence. It was clearly established, that before the alteration in the law respecting suicide, the fear of being buried in a cross-road, and having a stake driven through the body, had no beneficial effect in decreasing the number of suicides; and the verdict of *felo-de-se*, now occasionally returned, is productive of no advantage whatever, and only injures the surviving relatives.

"When a man contemplates an outrage of the law, the fear of the punishment awarded for the offence may deter him from its commission; but the unhappy person whose desperate circumstances impel him to sacrifice his own life can be influenced by no such fear. His whole mind is absorbed in the consideration of his own miseries, and he even cuts asunder those ties that ought to bind him closely and tenderly to the world he is about to leave. If an affectionate wife and endearing family have no influence in deterring a man from suicide, is it reasonable to suppose that he will be influenced by penal laws?

"If the view which has been taken in this work of the cause of suicide be a correct one, no stronger argument can be urged for the impropriety of bringing the strong arm of the law to bear upon those who court a voluntary death. In

* "The Anatomy of Suicide." One Vol. 8vo. By Forbes Winslow, M.D.

the majority of cases it will be found that some heavy calamity has fastened itself upon the mind, and the spirits have been extremely depressed. The individual loses all pleasure in society; hope vanishes, and despair renders life intolerable, and death an apparent relief. The evidence which is generally submitted to a coroner's jury is of necessity imperfect; and although the suicide may, to all appearances, be in possession of his right reason, and have exhibited at the moment of killing himself the greatest calmness, coolness, and self-possession, this would not justify the coroner or jury in concluding that derangement of mind was not present."

The verdict of *felo-de-se*, however, appears to have given general satisfaction, if we are to judge by the tone of the public press. We have only room for one extract on the subject, and that is from the *Christian Times.*

"The coroner's jury appointed to inquire into the circumstances under which John Sadleir met his death have returned a verdict of *felo-de-se.* The finding comes upon us with a startling effect, for verdicts of this stern character had become nearly obsolete. The spurious liberality, leading to a morbid humanity, which is the characteristic of our times, had rendered it almost a matter of course that, in cases of self-murder, the verdict of the coroner's jury should ascribe the act to 'temporary insanity.' We are glad that the Hampstead jury have taken a more manly view of the obligation imposed on them by their oath, and that they have not been deterred, by any fear of injuring the feelings of relatives or friends, from returning a verdict according to the evidence. We should be glad to regard this departure from the conventional and stereotyped form which has too long characterized verdicts in such cases, as symptomatic of a return to a more elevated standard of public morality, when men shall think more of duty and less of the consequences that may flow from its discharge.

"That John Sadleir took away his own life while in full possession of his mental faculties, will not, we think, be disputed by any one who read the evidence. Every circumstance attending the awful tragedy showed marks of forethought and deliberation. The stratagem practised to obtain the poison; the arranging his affairs, as far as such a hopelessly entangled web could be arranged; the selection of the scene of suicide, at a distance from the house which his brother's family and he jointly occupied, coupled with the precautions taken for the identification of his corpse; the feeble attempt to assert, even in that horrible hour, his position as a gentleman, by using a silver cream jug, out of which to drink the poison—a minute but significant stroke of character which throws all that Dickens has imagined into the shade;—all these tend to show that John Sadleir was of sound mind when he planned his own destruction, and retained his intellect till the last moment of carrying his fell purpose into effect. As this is the first case that has occurred for a long time where a jury has had moral courage enough to return such a verdict, so it must be added that there are few cases where every hypothesis of insanity has been more completely excluded. Every one who knew John Sadleir admits that he was a clever and active man of business, and the methodical habits which made him such clung to him to the last. Everything was scrupulously arranged; and it is affecting to observe that while so many matters of no moment were cared for and provided against, the same force of will was exercised in determinedly shutting out from view the more awful considerations of futurity.

"We would not, however, be understood to bear harshly on the memory of this wretched man. In truth, we have no heart to do so. Even the frauds he committed, gigantic as they are, lose their proportions when we contemplate the miserable tragedy to which they led. The letters which were written by him give us some slight glimpses of the agony which racked his mind during the last night he spent on earth; but after all, how frail and feeble must those glimpses be compared with the dread reality! Who shall attempt to gauge the keenness of that remorse which gnawed him as he sat alone, at midnight, with all the memories of the past rising before him in such unnatural brightness as to darken with the blackness of despair every prospect for the future? Who shall attempt to delineate that rush of fearful fancies through his mind in his ghastly death-walk along the silent streets to the yet more lonely hill! There is nothing more terrible than to behold a human spirit thus caught in the meshes which its own guilt has woven round it, struggling desperately for escape, and struggling in vain. It is rarely we are permitted to see acted visibly, almost before our eyes, the moral retribution of guilt thus consummated in this world. Would we could hope that the lesson might be as lasting as it is impressive!

"This terrible tragedy will not have been enacted in vain, if it shall lead society back to the great moral truth, that neither wealth nor station are the great ends of life; that the aspirations of ill-regulated ambition are ever in opposition to the dictates both of Revelation and experience, which emphatically proclaim that man's life has to do with the lowly rather than the high, with the common and the familiar rather than the brilliant and the startling; that the battle-field where true glory is to be won, lies not in the broad and vulgar light of this world's applause, but deep down in the secret recesses of the heart, where the strife and the sweat, the agony and the victory, can be discovered by Him alone who has promised to all who overcome in secret, that He 'will reward them openly.'"

In relation to questions of *felo-de-se*, we would direct attention to the following unique and singular case of suicide. What do our readers think of this man's mental state? Was the verdict of "temporary insanity" justifiable? It constitutes a melancholy illustration of the state of mind occasionally known to precede the act of self-destruction. Cases of suicide have been committed by persons upon whose bodies, after death, letters have been found earnestly begging the coroner to repudiate all attempts on the part of the jury and family to record a verdict of insanity! They have been anxious to "die game," and to escape the stigma of lunacy and irresponsibility, so strangely constituted is the human mind!

"An inquest was held by Mr. W. S. Rutter, county coroner of Lancashire, on the body of Mr. John Hobler, aged twenty-one, a young gentleman of highly respectable connexions in London, who had been studying the profession of a machinist and tool-maker at the works of Messrs. Whitworth and Co., of Manchester. The deceased resided with Dr. Clay, of Manchester, a relative, and absented himself in the month of January, leaving letters behind him stating his intention to destroy himself. Of course his absence created much alarm and uneasiness, and his friends advertised him, and took measures, through the police, with a view to discover his retreat, but in this were unfortunately not successful. On Wednesday his dead body was discovered in a copse or plantation called Cheetham's Close, in Turton, near Bolton, near a road but little frequented; and, from the state of decomposition it was in, the body had probably been there for some weeks. An empty phial and wine-glass were found near the body, leading to the supposition that the unhappy gentleman had poisoned himself. The body was discovered by a boy in company with Mr. Kay, of Turton Tower, the owner of the property, while in search of game. In one of deceased's pockets was found a letter with this address outside:—'The finder of this body, particularly addressed to the police, inquisitiveness being their misfortune.' The letter was as follows:—'*If they make it a case of temporary insanity, it will be so—on the part of the jury. Dearly beloved cove or coves, now that you have found this body, do not be an idiot or idiots, and get up an inquest to talk about what they know nothing about; but somewhere in my pockets you will find* 6*d*., *with which get a gill. After that, stick my body in a hole somewhere. You need not read over it any service, as I read it myself before starting; and by selling my clothes you will be able to pay yourself for your trouble. After which, go away: and above all things do not try to find out who I am, &c., as you will not be able, but do as I say, and the Lord reward you accordingly. Yours truly. The verdict must be that, in great consideration for some person or persons, he destroyed himself to save them the trouble.*' At the time the inquest was commenced the name of the deceased had not been ascertained, but Dr. Clay, of Manchester, afterwards identified the body, and the jury agreed to a verdict to the effect that the deceased had destroyed himself while labouring under temporary insanity. Dr. Clay stated that, before leaving Manchester, Mr. Hobler was in a low and desponding state of mind, brought on by over-study."*

Mr. Sadleir's sad case has been made the subject of comment and animadversion, not only in the pulpit, but in the principal journals of the day. The *Times* has not remained silent. With its usual sagacity, power, and brilliancy of illustration, it has endeavoured to draw an important lesson from Mr. Sadleir's eventful history and melancholy death. The observations of the Editor deserve to be universally read, and seriously studied by all interested in the moral condition of the nation, and anxious to preserve intact the stability of society. The vice of the age is undoubtedly covetousness; a craving, insatiable, and morbid appetite to obtain the *maximum* amount of wealth at the *minimum* expenditure of labour. This has led to reckless speculation; and on the heels of speculation have followed inevitable ruin, disgrace, and often premature death! The philosophical remarks of the *Times* are so replete with material for serious and philosophical thought, that we make no apology for quoting them entire.

"Death is the last punishment which the justice of man can award to the greatest criminal. When the moment of enforced agony is over, and the guilty spirit is dismissed to the judgment-seat above, there is an end of all vindictiveness and all animosity. There must, however, be no confusion of the eternal difference between right and wrong—no maudlin palliation of great crimes, because the best among us have often tripped and sometimes sinned. Let us at least abhor vice if we do not always avoid it. Let us, indeed, be sterner critics upon ourselves than upon our fellow-creatures; but no false commiseration or complaisance should bind us to silence when a country is startled with a rumour of a great crime because the criminal has expiated his guilt by a violent death. Our readers must have already perceived that we are speaking of the suicide of John Sadleir. It is not for the purpose of denouncing the individual, nor of giving additional publicity to his disgrace, that these observations are intended. The fact of his violent and horrible death, and the history of his crimes, are already so notorious, that nothing we can say could

* *The Times*, March 15th.

add to his dishonour. If we remark upon the tragical end of his career at all, it is as a protest against that fearful spirit of speculation which is the vice of our times; it is that others may see in his fate something akin to that which awaits themselves when they are driven to the end of their shifts and contrivances—when they have exhausted the suggestions of cunning and the resources of crime. The bankrupt's despair, the felon's cell, the cold bed of the suicide on the damp moor, must be reached at last, the fitting termination to a life only supported by the plunder and misery of others. John Sadleir does not stand alone in his guilt and in his shame. The criminal records of the last year can show the names of men who stood as high or higher in the world's eye, but of whom we now forbear to speak, from very pity for their fallen estate. Hundreds, we fear, of persons in this metropolis, and in the larger mercantile towns of the kingdom, are now engaged in the same perilous traffic. It needs but a reverse and an opportunity, and why should they hesitate to follow in the footsteps of the most keen-witted and unscrupulous of their predecessors?

"The proceedings of the adjourned coroner's inquest, which we publish this day, do not throw much additional light upon the nature of the embarrassments which drove John Sadleir to despair. Letters of his, written a few hours before he sallied out on his fatal expedition, were produced, but they inform us of little beyond what was already known. This is their general tenor:—'I cannot live. I have ruined too many. I could not live and see their agony. I have committed diabolical crimes unknown to any human being. They will now appear, and bring my family and others to distress, shame, and grief that they should ever have known me. I blame no one, but attribute all to my own infamous villany.' Such, as will be seen by our report of this day, is the general tenor of these letters, which are written in a spirit bordering on distraction. It is, however, sad to know that the self-accusations are not the mere suggestions of a diseased brain. It is true that the writer was guilty of diabolical crimes; it is true that he has swindled and forged to a fearful extent; it is to be feared—as he himself says—that he has been the cause of permanent misery to thousands of unoffending people. Let us not deceive ourselves, nor, out of false pity for the wretched author of all this sorrow, endeavour to palliate the atrocity of his acts. If the heinousness of crimes be measured by their consequences, the man who carries disaster, if not absolute ruin, into a hundred families, is stained with deeper guilt than the mere ruffian who attacks life. The report is that, when the full extent of his villanies is known, it will be found that, in one form or another, he has swindled the public to an amount little short of half a million. It would seem marvellous to those who have not been under the unfortunate necessity of watching the course of modern speculation, how a man who, probably, started in the world without any fortune at all, could involve himself and others to such an extent. In Sadleir's case, however, it must be considered that, independently of the ordinary resources of the speculator, he was enabled, from his official position, to commit many crimes without fear of immediate discovery, which another man could not have attempted without instant detection. It would be as yet premature to suggest any precise estimate as the limit of his defalcations. When a man is in a position to forge title-deeds, railway shares, and mortgages to an indefinite amount, there need be little limit to his operations. He is also charged with having fraudulently assigned away deeds held in trust by him to an enormous amount. As a forger, he seems to have been remarkably successful, and it is said that the signatures of the Irish Encumbered Estates' Commissioners have been so skilfully executed, that no one, however familiar with their handwriting, could distinguish between the forgeries and the genuine signatures. At the close of last week it was added that many forgeries on private individuals had been already made out, and that the discovery of many more was anticipated. On the whole, this seems the greatest crash made by any single individual in recent times.

"We purposely avoid dealing with what may be called the 'picturesque' features of this remarkable case. We have no wish to dwell upon the despair of the detected forger, and his last ghastly walk to the heath where he was about to lay down his life. Burglary and murder have been made 'interesting' ere this, but let nothing of this kind be attempted now. We would wish to hear the crimes of John Sadleir spoken of with wholesome and universal abhorrence, but let even indignation spare his unhonoured grave; let there be no morbid dwelling upon the last scenes of his life nor upon his closing agony. He has already appeared at the bar of that Almighty Judge before whom we must all of us one day stand; to that tribunal let him be left. If words of ours would avail, we would deprecate all further and unnecessary prying into the secrets of the family even by the coroner and his jury. It is surely proved that John Sadleir died by his own hand, being unable to bear the shame of exposure and the consequence of his crimes. What has the public to do with the distracted letters addressed by the suicide to his relatives in the last moments of his career? One sentence from these contains the whole moral of his guilty life and tragical death:—'*Oh! that I had resisted the first attempts to launch me into speculation!*' There are many of the English public who would do well to lay seriously to heart the dying words of John Sadleir."

We place upon record the particulars of two other remarkable cases of suicide. In one case a man destroyed himself *by deliberately thrusting a red-hot poker down his throat;* and in the second instance a person precipitated himself, in a state of delirium, from the whispering-gallery of St. Paul's Cathedral!

"On the 23rd Dec., at ten o'clock, a man in the prime of life, but whose appearance betokened poverty and misery, entered the Grantham Arms, Dyer-street, Leeds, and having called for a pipe, sat down moodily by the fire. Two or three persons were sitting in the room, but the stranger was not heard to speak a word. After sitting thus for ten minutes the man put a poker into the fire, and, when it had become red hot, took it out and knocked it against the floor to remove any excrescence on it; he then deliberately put the red-hot end of the poker down his throat. The persons present caught hold of him, and, having removed the poker from his possession, bathed his mouth with warm water. The man was ultimately removed to the Mendicity Office, where every attention was shown him; and, in answer to inquiries as to the cause of the rash act, he only replied that it was a very foolish act, and he did not know what he was doing. His tongue, throat, and under lip were very much burnt, from the effects of which he died last Friday. The only information that can be gained about the deceased is, that his name is Thomas Barker, and that he came from Bolton, in Lancashire. An inquest was held on the body at the Court House on the 30th Dec., before Mr. John Blackburn, when a verdict was returned—'That Thomas Barker died from the effect of the burns which he had wilfully inflicted on himself; but there was no evidence to satisfy the jury as to the state of his mind.'"

"A person named Alexander Smart committed suicide by precipitating himself from the whispering-gallery of St. Paul's Cathedral into the nave, a distance of nearly 150 feet. The deceased had been a clock and watch maker, but had retired from business a considerable time, and had been residing with his wife at 4, South-street, Berkeley-square. After he had waited in the gallery until the clock struck twelve, he mounted the handrail of the gallery, gave three hysterical laughs, and cried out each time, 'Ah, ah, ah!' Two vergers, named

Thompson and Hutchins, who were both in the gallery, anticipating what he intended, rushed forward, but before they could get to the spot he had succeeded in jumping from the handrail and fell upon the stone flags which pave the nave. The warders ran to the spot, when they found deceased lying on the pavement of the church, quite insensible. Mr. Keating and another gentleman were sent for and promptly attended, but upon examining the body, they pronounced life to be extinct. The deceased had received compound fractures of the left shoulder, the legs and one arm were broken, the spinal cord was dislocated, and besides other injuries the head was beaten in by the fall. Upon the body of the deceased were found several pieces of paper bearing his own address, a sovereign, a five-shilling piece, one shilling, sixpence, and a letter addressed to a well-known nobleman. It appears that on a former occasion the deceased shot himself at the side of his head, but did not receive any mortal injury."

We are glad to perceive that the important social question of public and popular amusements is occupying the attention of thinking men. Our readers will recollect that in our last number we cursorily referred to this subject, in connexion with our comments upon the proper observance of the Sabbath-day. The subjoined remarks of the Rev. Dr. Guthrie on the subject deserve to be universally read:—

"The love of excitement is so engraven in our nature that it may be regarded as an appetite. Like our other appetites, it is not sinful, unless indulged unlawfully, or to excess. It is the duty of patriotic and Christian men to restrain these within due limits, and direct them into innocent channels. Indeed, it would appear that God has implanted such a feeling in all his creatures, for the purpose, no doubt, of ministering to their happiness. Did you never see a kitten chasing its own tail? Were you never amused with that? Those who are shut up for life in large towns, and never see horses but in the yoke, nor any of the feathered tribes but a sooty, begrimed, and melancholy sparrow, may be ignorant of the habits and happiness of the lower animals; but who, accustomed to the country, has not seen the crows on a summer evening wheeling, chasing, and darting at each other in the blue sky overhead, and the trouts amusing themselves, much after the same fashion, in some glassy pool? I have seen a merry flock of kids disputing in great good humour which should hold the stump of a decayed tree, and without a blush, confess to having reined up my steed, and with the old greybeards—the grandfathers and great-grandfathers of the youngsters—who formed a delighted circle of spectators—I have stayed to see the play. And often last summer did I sit in my boat to watch the butting, capering, racing, dancing, of a herd of red deer on the shores of Lochlee, and never thought ill on that account either of them or of myself. I don't envy that man his heart who does not feel happy to see God's creatures happy, nor praises God for his kindness to the meanest of them. Now, to frown on this love of excitement and amusement, as if it were guilty and a sin, appears to me a reflection on Providence. I will not reject any gift which God has given, but take it thankfully, and try to use it well. Take the case in hand—the musical entertainments in Dunedin Hall—which, although their harmony has been followed by so much discord, I shall continue to support so long as they are conducted as they have been begun. If the devil gave man an ear for music, and the pleasure in music which those gifted with such an ear enjoy, then let the whole affair be denounced; but if this is a gift of God, let it be consecrated to His service in the church, and out of it also, by being used not only as a source of innocent, thankful enjoyment, but as a means of weaning or keeping ourselves or others from debasing or forbidden pleasures. This is a noble use to make of music; and I cannot take blame to myself either for the end I had in view, or for the means by which I sought to gain it, when I countenanced the entertainment in Dunedin Hall."

The Hon. Edward Everett, in a speech delivered at the "Webster Festival," held at Boston, U. S. America, thus refers to the importance of recreation. The orator, after alluding to Mr. Webster's tastes for manly sports, says—

"The Americans, as a people—at least the professional and mercantile classes—have too little considered the importance of healthful generous recreation. They have not learned the lesson contained in the very word which teaches that the worn-out man is re-created (made over again) by the seasonable relaxation of the strained faculties. The old world learned this lesson years ago, and found out (*Herod.* I., 173) that as the bow always bent will at last break, so the man, for ever on the strain of thought and action, will at last go mad or break down. Thrown upon a new continent—eager to do the work of twenty centuries in two—the Anglo-American population has overworked and is daily overworking itself. From morning to night—from January to December—brain and hands, eyes and fingers, the powers of the body and the powers of the mind, are in spasmodic merciless activity. There is no lack of a few tasteless and soulless dissipations which are called amusements, but noble athletic sports, manly outdoor exercises, are too little cultivated in town or country."

The great increase of late of brain disease, and anomalous affections of the nervous system may, to a certain extent, be traced to the excessive and continuous strain kept upon the cerebral functions in the efforts made to obtain a respectable social and professional position. The brain is exercised at the expense of the physical health; and whilst the mind is worked to the full extent of its powers, the muscular system is quite neglected.

During the past quarter many elaborate and ably-written criticisms have appeared in the provincial press of the first number of the new series of this journal. Many of these analytical notices are distinguished by a thoughtful and philosophic suggestive tone, reflecting great literary credit upon their authors. I have been much pleased with an able and well-written criticism published in one of the leading provincial papers, viz. the *Berwick and Kelso Warder*. It is gratifying to find men of a philosophic turn of mind, evidencing a taste for the higher walks of literature and science, officially connected with journals exercising in the provinces great influence upon public opinion. The writer of the article in the *Berwick and Kelso Warder* is Mr. WILLIAM GRAY. His remarks on the study of mental philosophy are so pertinent that we make no apology for placing them before our readers.

"Mental Philosophy is rapidly reaching the ground of the experimental, or inductive philosophy of Bacon. Three great names are conjoined in condemnation of the old philosophy of Scotland — and of the metaphysicians of the school of 'the pure reason,'—the names of Goethe, Chalmers, and Carlyle. What, asks Goethe, 'what is the use of thinking about thinking?' And, with Goethe, we pause for a reply. It needs the genius of such a man as Professor

Ferrier, of St. Andrews, to make such abstractions as he deals in readable at all. Instead of beginning with the insoluble problem of the relation of mind to matter, which is what is properly called metaphysics, we hold that the true starting point of mental philosophy is to ascertain, first of all, from consciousness, what are the indubitable facts of which our human *nature* is composed. Observe, we do not say—of which the human *mind* is composed—for here we believe has taken its origin that delusion which till recently led metaphysicians a wild-goose chase. We say, that if you wish to study the manifestations or phenomena of mind, you must, to be truly philosophical, take into your account that material frame in which that mind has its habitation and its home. You must study all phenomena of mind as if they also were corporeal, because they are indissolubly connected with a material organization. Here, we say, as in the rite of holy matrimony, and with no irreverence, what God has joined together, let not man put asunder. The phenomena of mind are to be studied thus in the concrete, in all their relations to, and manifestations in connexion with, that physical frame which is so fearfully and wonderfully made, that it is evidently intended to be studied by the mental philosopher as well as by the anatomist. The authoress of 'Jane Eyre' has said, in one of her poems, which are quite as wonderful as her novels, that all the world, with its goodly furniture of objects, existed as a thought in the mind of God subjectively, before it took the objective shape of the fair creation we behold. And divines have traced from that same work certain mental attributes reversing the process of the poetess, whose fine idea we commend to Mr. Gillespie, and the affirmers of the reality of an argument for the being of a God *à priori*. Now, if the world may be regarded as the expression of God's thought or mind, why may not man's material frame be the outward clothing of the mind, and if such a presumption be not absurd, why should we not study the outward demonstration conjointly with the vital force within? Farther, it is well known that the study of the anatomy of the human frame remained almost stationary, until it received a new impetus and impulse from the illustrations given to it by comparative anatomy. That is to say, those inquirers after truth, who were in the habit of carrying their researches by dissection into the framework of all kinds of animals, were arrested by some striking peculiarity which led them to examine further whether or not it might not cast some new light on some mysterious portion of human organization. And they found that it did so, and hence arose the necessity of comparative anatomy to the proper investigation of human anatomy. Now, if the comparison of the physical framework of what is called the lower creation with that of man helps to the right understanding of the latter, why, we ask, may not the examination of whatever intelligence is possessed by the lower animals, and the comparison of it with man's intelligence, cast a new light upon the latter? We do not believe that the lower animals have *souls*, but we believe firmly that they have *minds*, or intelligence. A soul is a moral, and therefore accountable being, as well as an intellectual one; but a dog, for instance, although not a moral and responsible being, may yet have some intellect. This distinction will save us from being identified with the philosophers of the materialistic type represented by the notorious 'Vestiges of Creation.' We do not wish to degrade man, but we wish to do justice to the dog, and the horse, and such noble representatives of the lower, inarticulate world. We have in our own possession a little Scotch terrier, which gives as unequivocal tokens of intelligence as can possibly be. And the stories told of the intelligence both of the dog and the horse are endless. Now, all this new ground, with many investigations on the connexion with man's frame of light, &c., has been but lately broken up. Perhaps we owe something of the direction of modern thought to this mode of treating mental philosophy."

The past quarter has been marked by the average number of medicolegal inquiries. There have been *four* trials in which insanity has been urged as an extenuating plea. We place them in chronological order:

*Mary McNeill*, 25, spinster, for the wilful murder of George McNeill, her son. Found not guilty, on the ground of insanity.

*Sarah Allen*, 33, married woman, for the murder of her two children, William Allen and Arthur Joshua Allen. Found not guilty, on the ground of insanity.

*Thomas William John Corrigan*, 29, for the murder of Louisa Corrigan, his wife. Guilty. Sentence of death passed.

*Emily Rider*, 21, married, for the murder of Frank Withers Rider, her son. Found not guilty, on the ground of insanity.

*Charles Broadfoot Westron*, 25, for the wilful murder of George Waugh. Found guilty, but recommended to mercy on account of his strong predisposition to insanity.

Mary McNeill was tried for cutting the throats of two of her children on the 28th of November last. In this case there does not appear to have been any obvious exciting cause for the crime. She is said to have been very comfortable and happy with her children up to a short time antecedent to the murder. A change was then observed in her appearance. She became melancholy, and was frequently heard to moan about her children. After her last confinement she had an attack of "milk fever." This was succeeded by an obvious mental change. It was proved that her father was in confinement as an incurable lunatic. Her homicidal symptoms had been a matter of observation for some time prior to the commission of the murder. The prisoner's third boy was sent from home in consequence of her having been seen to hold him over the balusters, exclaiming, "I'll drop him; I'll drop him!"

The case of Sarah Allen was, in its main features, similar to the preceding. This wretched woman imagined that her children were suffering from scrofula, and that her husband had the same disease. This preyed much upon her mind. She was heard to exclaim, "My husband is ruined; my children are ruined: we are a ruined family!" The prisoner and her husband lived happily together, and she was said to be passionately fond of her children. It appears that she threw the two children into the water near the Cadogan Pier, Chelsea, one very foggy night, asserting, on her return home, that she had lost the children in the fog.

"Mrs. Richards deposed that she had known the prisoner a long time. She says:—She came to my residence in Cirencester-place, Fitzroy-square, on the evening of the occurrence. It was about seven o'clock when she came. She was alone, and appeared to be very desponding and in great distress. I hardly knew her at first. She said that she wished to speak to me, and then she added, 'I have lost my children.' She was crying. I told her to come upstairs, and inquired where her husband was; and she said she had not seen him since morning. I then told her that she must go to her husband directly, and she said, 'No, I cannot go back.' I then told her that whatever might have happened she must go to her husband, and that he was her best friend. The prisoner kept repeating that she could not go to her husband, and it was two o'clock in the morning before she consented to go home, and I then accompanied her to her husband's house. The prisoner gave me no account of what had taken place, but merely stated that she had lost her children. When we got to the door of her house the prisoner took out a key and let herself in, and I told her to go upstairs and see if her husband was at home, and she did so; and she knocked at her husband's door, and he let her in. When he saw her, he said, 'Sarah, it cannot be you;' the prisoner said, 'Yes, it is,' and she added that I was downstairs, and I went up and saw one of the children lying in the bed, and the prisoner took him up and kissed him, and said, 'Where did you get him from?' The prisoner's husband then said, 'Where is Willy?' and she made no reply. He then asked where the baby was, and the prisoner said, 'Where is my baby? Why cannot I have my baby?' The baby was at this time in the charge of a nurse. The prisoner repeated the words, 'Where is my baby?' a great many times. The prisoner appeared in an agony of grief when she came to my house, and I never saw her in such a state before."

Dr. Rowe, and Mr. Gibson, the surgeon of Newgate, gave medical testimony in the case:—

"Dr. Rowe (of Cavendish-square) had visited the prisoner since she had been in Newgate, and found that she was still under the delusion that her children were suffering from scrofula, and she told him that their foreheads projected, and that their eyes were starting from their heads. He reasoned with her, but it had not the least effect. He had no doubt that the delusion had been very much strengthened by the perusal of the book that had been mentioned. A delusion of this kind was very common among persons of a nervous temperament, and, although it would sometimes disappear, very great care and long treatment were generally required to obtain that result. The prisoner told him that she considered her children were better off now they were dead. A delusion of this character was very likely to induce the commission of such an act as the one now charged against the prisoner.

"Mr. Gibson confirmed the evidence of Dr. Rowe, and also stated that it was his opinion that if the prisoner had thrown the children into the water she was incapable at the time of distinguishing between right and wrong."

The case of Corrigan stands next in rotation. It appears that this wretched criminal brutally murdered his wife whilst under the influence of intoxicating drink. He had previously suffered from attacks of *delirium tremens*, and was represented to be an habitual drunkard!"

A witness, Anne Burton, deposed as follows:—

"On the day of the murder she observed that when the prisoner came first he appeared in good spirits and very happy. They drank gin-and-water and brandy-and-water during Christmas-day, and when witness and Mrs. Fearon went to bed they left the men drinking. About eleven o'clock at night she observed that the prisoner looked very excited. She saw him again on the following morning. He was then dressed and ready for business, and he drank two cups of cocoa hastily and left the house, and he said before he went that he did not feel very well. She saw him again at her house about half-past two o'clock, and he was then very much intoxicated. He went upstairs and returned in about a minute, and he then left the house. About four o'clock in the afternoon he came again, accompanied by her sister and the deceased, and they appeared to be talking cheerfully together. In a very short time witness heard a faint shriek, and aftewards very violent ones, from the bedroom, and she ran there, and some one passed her hurriedly. When she got into the bedroom she saw the prisoner lying on the child's bed with an open knife in his hand. She ran to him and laid hold of the knife; he struggled violently with her and threw her from him, and she screamed for help, and asked the prisoner if he wanted to do her any harm, and he made no answer. She said to him, 'What have you done?' and he looked wildly round, and made no answer. Her husband and Mr. Mahony then came to her assistance, and took the knife from the prisoner, and she locked it up. She had heard that the prisoner and his wife lived unhappily, but she knew nothing about it herself."

No attempt was made to establish ordinary insanity or delusion in this case. The defence was based entirely on the fact, that Corrigan was a sot, and that he murdered his wife whilst in an irresponsible state of mind caused by partial intoxication. The medical evidence in this case was extremely unsatisfactory, and certainly not sufficiently conclusive to justify the acquittal of the prisoner. Corrigan appears to have acutely felt his position after the murder of his wife. The following letters were written to his relatives during his confinement in Newgate:—

"House of Detention, Friday afternoon.

"Dear Betsy,—With a broken heart I write you to take all care you can of my poor dear children till I can make some arrangements with my friends. Do not pay any rent out of that trifle I left you. Please God they will be able to get up a benefit for me at the theatre or some place, and I expect there will be 6*l.* allowed for the funeral, You must get it done as cheap as possible, but do not slight the remains of my poor murdered wife. Oh! Betsy, if you knew the anguish of my mind. I have no rest night or day now that I have come to my senses. Oh! Betsy, save me a lock of my poor Louisa's hair. Now that she has gone I would give anything to undo what I have done. Be kind to my poor helpless children, and the great God that I trust to for mercy for my crime will reward you. Oh! Betsy, forgive me for what I have done, and beg of your father to do so too. None of your feelings, bad as they are, can be like mine, as I am the cause of all. If you cannot come to-morrow, you cannot come till Monday, when, if you would bring a little butter with you, I should be glad. Give my love to my poor father and sisters, and accept the same yourself from your heart-broken, wretched brother-in-law,

"T. Corrigan.

"You must try and come at twelve o'clock."

"House of Detention, Friday night.

"My dear Mrs. Fearon,—In the midst of my dreary solitude I write to you to beg you to forgive me for the injuries I inflicted on you in my madness of Wednesday, though I earnestly feel assured that you believe they were accidental. But, my God! if I had wounded you in a vital part as well as my wife, what would have been my torture to know that I had committed two murders! Let me beg of you to forgive me for the horrors of body and mind I have caused you, and also Anne and your sister. Tell them I hope their fate will be happier than mine. Give my kindest and best respects to them both. Ask them not to hate me. Tell them that I would not deliberately hurt a worm, either drunk or sober, much less go so bloodthirstily to work as to buy the knife in cold blood to murder a woman. Little did I think when I bought it, what would become of it; but it is done, and may God have mercy upon me for it! Give my respects to Tom and Dan, and all who inquire after me, and thank them for their interest in my behalf. If you have an opportunity, can you see Jack, and ask him to exert himself about a counsel for me as soon as possible? I have written to Ben Rose. I have never been very backward in assisting others, and among them all something may be done; but don't have ———, have somebody better than him; have a counsel for criminal cases, and send him to me before Thursday, so that he may watch the case at Arbour-square on Thursday morning if possible. I will endeavour to collect my energies together, and pray to God to give me strength to go through it all. Pray for me, too, all of you, for we none of us know what is over our heads. Who would have thought on Christmas night that I should have been here now? Good-bye, God bless you, and Tom, and Anne, and Louis, and Dan, and all of you. That God may take you all into His good keeping is the earnest and heartfelt prayer of

"Your faithful and true friend,
"T. Corrigan."

Corrigan, after an impartial trial, was condemned to death. He was subsequently reprieved by the authority of the Secretary of State, on the ground of his not being, at the time of the murder, in a sane and responsible condition of mind. He is not, however, to be exempt from penal servitude, by being sent as an insane criminal to a lunatic asylum. This is as it should be. It would be perfectly monstrous if such a class of criminals were permitted to escape the clutches of the law on such unsatisfactory and unscientific grounds. Human life would not be safe if ordinary temporary drunkenness and culpable sottishness were to be considered as a legal excuse in cases like that of Corrigan's.

Many good and humane men are of opinion that intoxication, instead of being a justification, should be viewed as an aggravation of offences committed whilst in this voluntarily-induced condition of temporary mental aberration. "*Ebrietas*," says Seneca, "*nihil aliud est quam voluntaria insania*." By the Grecian law, a double punishment was awarded for crimes committed during fits of intoxication. It was considered necessary not only to punish the crime, but also the drunkenness that originated it. The Roman law took a more lenient view of the subject. The plea of drunkenness was considered a valid one, except in the case of females; these it subjected to capital punishment. The subject of crimes committed during fits of intemperance is not mentioned in the French code, but it has been decided by the highest judicial authorities in France, that drunkenness being both voluntary and reprehensible, can never be advanced as a legal excuse for crime; such is also the law in Austria, Germany, and England.

The crime of Westron for the murder of Mr. Waugh, in Bedford-row, is one different in its main features from those previously referred to.

This was a case of premeditated murder, which Westron for some time had conceived and determined upon, and which he coolly and deliberately carried into effect! Westron had threatened Mr. Waugh's life a few months before he shot him, and fearing that he would commit some act of violence, Mr. Waugh summoned him before a magistrate, and bound him over to keep the peace. It appears that Westron had employed Mr. Waugh to act as his solicitor in the recovery of some property to which he and other members of his family considered they were entitled. Mr. Waugh had, after much difficulty, succeeded in his suit. Westron, however, thought Mr. Waugh's bill of costs very exorbitant, and not being able to obtain a sufficient advance of money from Mr. Waugh, Westron became much irritated, and threatened to commit acts of violence. Mr. Waugh had agreed to advance Westron a sum of money, on account, on a particular day; but, in consequence of Mr. Waugh's being obliged to leave London for Dublin, the promise was not fulfilled. This galled Westron very much; and under the influence of excited and irritated feeling, he waylaid Mr. Waugh in Bedford-row, and shot him through the heart! His death was instantaneous. After being arrested, far from expressing any contrition for his offence, he appeared gratified at the announcement of Mr. Waugh's death! The defence in this case was insanity. The following were the facts deposed to by the witnesses, and considered to be symptomatic of Westron's mental aberration:—maintaining that a bed six feet long was too short for him to sleep upon;—that he was frequently observed to talk loudly to himself by night and day;—parties were obliged to speak three or four times to him without getting an answer;—excitement, and using bad language;—conversation frivolous and childish;—used to swear terribly, and thump violently on the table;—was in the habit of throwing the furniture about;—asked for spirits to make a fire to burn the devil, who, he said, was always near him;—wanted a flat-iron to fight the devil with;—wished to obtain some bullets for the purpose of shooting the devil;—wanted to hatch some chickens by steam;—was in the habit of running up and down stairs during the night;—used to make his tea in a narrow mug, and said that if his friends came after him he would shove them into the gutter;—frequently sat for two or three hours without saying a word;—made a noise like a dog;—frequently looked up the chimney, and said it was too large, and that there was too great a draught;—complained of the doors and the keyhole;—frequently dragged the bed about the room;—when asked for a reference by the party of whom he was engaging a lodging, exclaimed, "Reference, that is —— humbug—I never give a reference;" —&c. &c. It was upon absurd facts like those just recorded that an attempt was made in Westron's case to bolster up the plea of insanity!

It is much to be regretted that any respectable physician should have gravely countenanced such a farcical proceeding. The jury, however, took what they conceived to be a humane view of the case, and recommended Westron to mercy "*on account of his strong predisposition to insanity!*" What a mockery of justice! If capital punishment was ever justifiable, it was so in the case of Westron. No valid excuse could be urged in his defence. If mere eccentricity—the use of bad language—refusing to give a respectable reference—looking up a chimney in a mysterious manner, having one or two relatives insane, are to be considered by medical men as scientific and conclusive evidence of lunacy, then the plea of insanity should be altogether abolished. Westron asserted that Mr. Waugh had "ruined and robbed him of his property." The editor of the "*Express*" has so fairly analysed the matter, that we make no apology for quoting at length from his critical comments on the case:—

"Now, was this an absolute delusion?—We do not say, was it entirely such a judgment as a cool and uninterested person, having all the facts of the case before him, would have formed? but, was it a mere figment of the man's fancy—a wild hallucination, having no basis whatever in fact? Most assuredly not. The murdered man was the lawyer of the murderer and his family, engaged on their behalf in managing certain family estates. The murderer wanted to sell his share and go abroad with the proceeds. Difficulties had been interposed—quarrels ensued. As long ago as last October the lawyer brought his client before a magistrate, swearing that at that time his life was in danger from his violence. An arrangement was then proposed and acceded to, under which Westron ceased to employ Waugh, and put his affairs into the hands of another solicitor, Mr. Sandys. Now, mark what follows. On the 14th of January, two days before the murder, Mr. Sandys, acting under Westron's instructions, had prepared a conveyance for the sale of his client's share in the family estates. *On that day* Waugh *prevented the completion of the purchase by insisting on deducting from the purchase-money certain charges, which had before been subjects of dispute between* Westron *and himself.* Westron, on learning this, became excessively indignant, and two days after shot Waugh dead under the circumstances with which all are familiar. When taken to the police-court fresh from the murder, Westron told the inspector—what? Why, that "if it had not been for Waugh he should have had 800*l.*, but now he should only have 400*l.*, and God only knew when he should get that, as it had been thrown into Chancery." Now, was this a delusion, a fancy, the groundless chimera of a morbid brain? Nothing of the sort. It was merely a view, by no means highly coloured or exaggerated, of an actual matter of fact—a view which, acting on a wicked and a malicious mind, impelled the man to a frightful act of deliberate murder. There being then no particular morbid delusion as to the relative position of himself and his victim, to reduce this act below the guilt of murder, an attempt was made to show that the assassin was, generally, a person of unsound mind. As far as we can gather from the evidence, this attempt entirely broke down. Taken altogether, it amounted to nothing more than this, that Westron was a wayward, moody, and somewhat savage being, irregular in his habits, and taking a decided pleasure in frightening the minds of weak landladies by theatrical displays of ferocious brutality. One of the doctors called to prove his insanity went to see him in Newgate; the man told him the same story he had told the inspector, and then forsooth began muttering something in a confused way about 'certain parties,' and 'certain other parties'—*argal*, quoth the doctor, he was mad.

The jury, after deliberation, brought in this extraordinary verdict: 'Guilty of wilful murder, but we recommend him to mercy on account of *his strong predisposition to insanity.*' More extraordinary still, Mr. Justice Wightman, after conferring some time with Mr. Justice Willes, is reported to have told the prisoner, 'that the jury had come to the conclusion that although he might be insane on some points, yet that he knew right from wrong, and they recommended him to mercy. Under these circumstances, he should abstain from passing sentence, and merely order judgment of death to be recorded.' Now, with all possible respect for Mr. Justice Wightman, we cannot think this right. Surely, he ought to have taken the opportunity of repressing at once the notion that a mere 'predisposition to insanity' ought to excuse from punishment in a case where the act was not committed under the direct influence of any morbid delusion. And this he might have done in the usual way, by informing the jury that he would forward their recommendation to the proper quarter, at the same time holding out no hope that the recommendation would be attended to. We regret that this course was not pursued; for it is impossible not to apprehend the introduction of a new element of uncertainty into the administration of our criminal law from the admission of a ground of mitigation so vague and unsatisfactory as 'a predisposition to insanity.'"

If Westron was a dangerously insane person, steps should have been taken to protect the public from his acts of murderous violence. Dr. Semple has entered fully into this question in some able communications published, since the trial, in the *Medical Times and Gazette*. He forcibly points out the necessity not only of revising the law of Lunacy in relation to cases of alleged insane criminal irresponsibility, but of protecting the public against the acts of persons who are permitted to be at large in an obviously dangerous state of mental aberration. The law should be very stringent on this subject, and the relatives of insane persons ought to be considered, to some extent, as responsible, if they knowingly and recklessly jeopardised the public safety by allowing a dangerous lunatic to be at liberty.*

Since the publication of our last number, Mr. M. Noble Bower has been appointed resident medical superintendent of the Stafford County Lunatic Asylum, and Mr. John Forster, editor of the *Examiner*, and author of the "Life and Times of Oliver Goldsmith," the "Lives of Statesmen of the Commonwealth," and other works of a high literary reputation, has succeeded Mr. Lutwidge, as secretary to the Commissioners in Lunacy. We congratulate our readers upon this appointment. Mr. Forster will assuredly prove himself to be the "right man in the right place." His reputation as a scholar and a man of letters, as an enlightened politician and an original and bold thinker, is fully established; and we are much mistaken if his knowledge of practical matters of business will not be found fully equal to his position. He brings to his present office (which we hope is but a stepping-stone to one more in unison

* Westron is to pass his life in penal servitude.

with his literary accomplishments, legal knowledge, and rare abilities) a manly, independent, and honest spirit. He may find a field for the exercise of these excellent qualities of head and heart in the position he now occupies.—We should be doing an act of injustice to a distinguished philanthropist and a well-known literary gentleman,* if we closed our retrospect without doing homage to the genius of Henry Mayhew, by referring to his praiseworthy attempt to call public attention to the sad condition of a section of our criminal population. The meeting that he recently convened of the "Ticket-of-leave men," was a significant, an important, and a deeply interesting sign of the times. We hope that this humane and Christian move in the right direction will meet with the encouragement and support it so richly deserves. The condition of the poor criminal after the expiration of his sentence and punishment is indeed a sad and melancholy one. He enters the world again with the mark of Cain upon his forehead. The fact of a man having once been a convict is tantamount to his social annihilation! Who will be bold enough to admit him as a servant into his house? who will tender to him the right hand of fellowship? who will dare to brave the censure of the world by employing a convict, or connecting himself in any way with a person (whatever may be his reformed character) who has just returned from penal servitude, or been discharged from the hulks? Why should not these men have an opportunity of regaining a social position, and of earning an honest livelihood? Why should they be spat upon and spurned like dogs from our doors, and be considered as objects of contamination and reproach? God knows that apparently the best and fairest of his creatures are often, in heart, the worst of criminals. It is no credit to a man "clothed in purple and fine linen, and faring sumptuously every day," *and who comes from a good stock*, to say that he is not a thief or a burglar. He is not driven by starvation to commit an offence against the laws; he has no wife or children crying piteously to him for food; he is fitted to resist temptation when exposed to it; his position, his early training, his social ties, his wealth, his education, all tend to keep him in the right path. Let us not permit ourselves to be scared or turned aside from the paths of mercy and active benevolence by the cuckoo cry of "maudlin sentiment for the criminal." It is too commonly the practice to throw this stereotyped phrase in the teeth of those who express any Christian and manly sympathies for an erring criminal brother. But good men and true of heart like Mr. Henry Mayhew, will not easily be frightened by such bugbears from carrying fully into effect his benevolent scheme. We wish him God speed in his noble work of practical philanthropy.

* Author of "London Labour and London Poor."

We were prepared to enter at length into an analysis of the important judgment of Sir John Dodson, in which that learned judge pronounced against the will of Mr. Dyce Sombre on the ground of insanity; but we consider it but fair to all parties to defer the publication of our remarks on the matter pending the hearing of the Appeal from the Prerogative Court to the Privy Council. In the mean time, as a matter of interest and curiosity we publish a list of the medical witnesses who gave their opinions *pro* and *con.* in this case. We confine ourselves to those gentlemen whose evidence was taken by the Court, and referred to by the Judge.

FOR THE INSANITY.

ENGLAND.

Sir James Clarke, Bart., M.D.
Dr. Conolly.
„ Elliotson.
„ Drever.
„ Munro.
„ Bright.
„ Southey.
Mr. R. Martin (Surgeon).

AGAINST THE INSANITY.

ENGLAND.

Mr. P. Blackwood (Surgeon).
Dr. Copland.
„ Ferguson.
„ Mayo.
„ Winslow.
„ Paris.
Sir Alexander Morrison, M.D.

FRANCE.

Sir Robert Chermside.

„ Joseph Olliff, M.D.

Dr. McCarthy.

„ Shrimpton.

„ Sigmond.

„ Béhier.

„ Ricord.

„ Bormeau.

BELGIUM.

„ Guislain.
„ Vlemmicke.
Baron Sentin.

There must be something seriously rotten in the condition of judicial psychology, when men of great experience and high position in this department of science come to such opposite conclusions from a consideration of the same facts. In connexion with the conflicting medical opinions expressed as to Mr. Dyce Sombre's testamentary capacity, the public will be disposed to exclaim,—

"Who can decide when doctors disagree,
And learned casuists doubt like you and me?"

23, *Cavendish Square*,
*April*, 1856.

